# Electrospun Nanocomposites Containing Cellulose and Its Derivatives Modified with Specialized Biomolecules for an Enhanced Wound Healing

**DOI:** 10.3390/nano10030557

**Published:** 2020-03-19

**Authors:** Marta A. Teixeira, Maria C. Paiva, M. Teresa P. Amorim, Helena P. Felgueiras

**Affiliations:** 1 Centre for Textile Science and Technology (2C2T), Department of Textile Engineering, University of Minho, Campus of Azurém, 4800-058 Guimarães, Portugal; martaalbertinateixeira@gmail.com (M.A.T.); mtamorim@det.uminho.pt (M.T.P.A.); 2 Department of Polymer Engineering, Institute for Polymers and Composites/i3N, University of Minho, Campus of Azurém, 4800-058 Guimarães, Portugal; mcpaiva@dep.uminho.pt

**Keywords:** cellulose, cellulose acetate, nanocellulose, antimicrobial surfaces, tissue regeneration, nanofibrous dressings

## Abstract

Wound healing requires careful, directed, and effective therapies to prevent infections and accelerate tissue regeneration. In light of these demands, active biomolecules with antibacterial properties and/or healing capacities have been functionalized onto nanostructured polymeric dressings and their synergistic effect examined. In this work, various antibiotics, nanoparticles, and natural extract-derived products that were used in association with electrospun nanocomposites containing cellulose, cellulose acetate and different types of nanocellulose (cellulose nanocrystals, cellulose nanofibrils, and bacterial cellulose) have been reviewed. Renewable, natural-origin compounds are gaining more relevance each day as potential alternatives to synthetic materials, since the former undesirable footprints in biomedicine, the environment, and the ecosystems are reaching concerning levels. Therefore, cellulose and its derivatives have been the object of numerous biomedical studies, in which their biocompatibility, biodegradability, and, most importantly, sustainability and abundance, have been determinant. A complete overview of the recently produced cellulose-containing nanofibrous meshes for wound healing applications was provided. Moreover, the current challenges that are faced by cellulose acetate- and nanocellulose-containing wound dressing formulations, processed by electrospinning, were also enumerated.

## 1. Introduction

Skin is the largest and outermost organ that covers the entire body, forming 8% of the body weight [1]. It is responsible for the body physical protection and sensitivity, serves as barrier to microbial and UV radiation, and regulates biochemical, metabolic, and immune functions, such as temperature, water loss (preventing dehydration) and synthesis of vitamin D3 [2,3]. 

When the skin barrier is disrupted through wounds, a series of complex physiochemical processes take place in an attempt to repair and regenerate the damaged tissue [4]. Wound healing is based on a complex series of cellular and biochemical processes, starting with inflammatory reactions (immune response to prevent infection), followed by proliferation (regeneration of tissues), and finalizing with tissue remodeling [5]. Based on the time that is required for wound healing, two types of wounds can be established: acute and chronic. Acute wounds usually heal within eight to 12 weeks after injury, while chronic wounds that include diabetic, pressure, and venous stasis ulcers, are unable to follow the normal healing steps, taking more than three months to heal. This inability to heal in a predictable amount of time occurs due to local (e.g., trauma, infections, radiation) and systemic factors (e.g., genetic disorders, diabetes, old age, smoking habit, vitamin deficiencies). However, in most cases, the presence of bacteria and the development of infections are the main causes [2,6,7,8,9]. Wounds are often colonized by *Staphylococcus aureus* (*S. aureus*)*, Escherichia coli* (*E. coli*), and *Pseudomonas aeruginosa* (*P. aeruginosa*) bacteria, and approximately 60% of chronic wounds display biofilms hindering their treatment. Biofilms are complex structures, which are formed of multiple groups of bacteria, often with different genotypes, which are further held together by extracellular polymeric substances (EPS). Their presence induces an immune response from the host. Local bacterial infections not only increase patient discomfort and delay wound healing, but they may also lead to more severe systemic infections. These microorganisms are responsible for high mortality rates in developing countries and have become an increasing cause of death in severely ill hospitalized patients, turning into an important economic burden in the health care system [10].

The first modern wound dressing was produced in the mid-1980s and it was characterized by its ability to maintain a moist environment and absorb fluids, and by playing a vital role in minimizing infection and promoting wound healing/management [11]. Modern dressings have evolved since then, being now recognized as interactive and bioactive solutions that combine the physical protection of traditional dressings with the ability of specialized bioactive molecules to stimulate cell regeneration through the proliferation and migration of fibroblasts and keratinocytes, to increase collagen synthesis, fight bacterial infections, and contribute with drug delivery functions for an efficient healing process. An optimal dressing is thus defined as capable of maintaining high humidity at the wounded site while also removing excess exudates, is non-toxic or allergenic, allows for oxygen exchange, can protect against microorganism invasion, and it is comfortable and cost effective. Modern dressings are designed as vehicles to deliver therapeutic agents at the wounded site, while assuming the most varied forms, including hydrogels, films, sponges, foams, and, more recently, nanofibrous mats [12,13,14,15]. 

Nanofiber-based dressings have attracted much attention in the fields of biomedicine, tissue engineering, and controlled drug delivery because of their intricate architecture. Dressings assembled while using nanofibers, produced via electrospinning, have shown clear advantages over conventional wound dressings. They resemble the morphological structure of the extracellular matrix (ECM) due to their nanoscale features, easily incorporating biomolecules or nanoparticles of interest, high porosity and large surface area [16,17]. In addition, these electrospun wound dressings have also shown good hemostasis, absorbability, and oxygen permeability, which are determinant factors for a fast and successful wound healing [18]. Various natural and synthetic polymers have been used in the production of polymeric nanofibrous mats via electrospinning, for prospective wound healing applications [19]. However, nowadays, there is a great demand for materials that are more sustainable, environmentally friendly, and capable of being processed at the nanoscale. When considering this, biomass-based polymers, such as cellulose and its derivatives, have become the hotspot of science due to their intrinsic properties. Cellulose, being one of the most abundant natural polymers on Earth, with relatively easy extraction, superior biocompatibility, non-toxicity, and biodegradability, has been considered as a factual option for wound dressings formulations, either as an additive or as base substrate. Acquired data has been very promising, with excellent effects being registered in regard to cell adhesion and growth [20,21]. However, the production of natural cellulose-based nanofibers, regenerated cellulose nanofibers, and even microfibers via electrospinning remains a very challenging process, due to their inability to dissolve in water and common organic solvents. In fact, most of the cellulose-containing nanofibers have been produced after extensive tests with a variety of solvents or by combining cellulose with other materials, e.g., polymers, metals or ceramics, and loading those formulations with bioactive molecules, such as drugs and growth factors [20,22]. The introduction of chemical groups within the structure of cellulose has facilitated processing and contributed for the emergence of cellulose derivatives, like cellulose acetate (CA), which is the most common derivative that is considered by the European Committee for Standardization (CEN) as a bio-based polymer [23]. CA is a polymer that is easily soluble in common organic solvents, such as acetone, acetic acid, *N,N*-dimethylacetamide, and their mixtures, low-cost derivative of cellulose with excellent biocompatibility, high water adsorption capacities, good mechanical stability, non-toxicity, and can be efficiently processed into membranes, films, and fibers from either solutions or melts [21,24,25,26,27,28]. Electrospinning allows for the production of CA-based nanomeshes with an intricate and complex architecture that can be functionalized with active biomolecules to address the specific demands of acute and chronic wounds via simple, reproducible and cost effective approaches [29,30,31]. The nanocellulose is another cellulose based material that has gathered much interest in the last few decades, for prospective biomedical field. Its biocompatibility, nontoxicity, biodegradability, water absorption capacity, optical transparency, and good mechanical properties have attracted researchers from all fields. Indeed, its incorporation in electrospun nanocomposites has contributed significantly for the overall composite increased mechanical properties, namely Young’s modulus and elongation at break [32,33,34]. 

When considering antimicrobial resistance one of the increasingly serious threats to human and animal health worldwide, there is an urgent need for more effective and target-directed therapies [35,36]. The present work provides an overview of the most recent dressing formulations containing natural-origin celluloses for chronic wound care. Their extraction, treatment, and further compatibility with other polymers were examined and their implications and potential to overcome these microbial threats was analyzed. The challenges in processing cellulose, cellulose acetate, and nanocellulose nancomposites via electrospinning were also highlighted. 

## 2. Nanostructured Wound Dressings

Traditional dressings, which are also known as passive dressings (simple gauze or gauze-cotton composite dressings, available since the mid-1970s), have as main function the protection of the wounded bed from further harm or to serve as barrier against the external environment; not however treating the wound or preventing bacteria from colonizing the site [37,38,39]. In an attempt to prevent these threats, modifications to the dressings’ structure have been proposed, resorting to either the grafting of non-adhesive particles at the inner surface or by using antimicrobial polymers, inorganic nanoparticles, or biomolecules [40]. These modified dressings are known as interactive or bioactive. Interactive/bioactive dressings can be defined as dressings with the capacity to alter the wound environment, optimizing the healing process. This group includes films, foams, hydrocolloids, alginates, hydrogels, and collagen-, hyaluronic acid-, and chitosan-based dressings, which stimulate the healing cascade [41,42]. Modern dressings, beyond protecting the affected area, should generate an environment conducive to healing, by:

▪ guaranteeing breathability;
▪ maintaining a suitable physiological temperature;
▪ ensuring a balanced moist environment, avoiding dehydration and cell death;
▪ promoting debridement;
▪ allowing proliferation and migration of fibroblasts and keratinocytes, and an enhanced collagen synthesis;
▪ protecting the wound from bacteria and other external soiling; and,
▪ adapting to the wound shape, without adhering [43,44,45].

Prior to the selection of the ideal dressing, health professionals should consider a number of parameters. Firstly, if there is necrotic tissue, debridement is required. Dead and decayed tissue in the wound area are critical, as they impede the healing process by stimulating bacteria proliferation, prolonging inflammation, and preventing reepithelization. Still, the healing process may be rekindled if conveniently cleaned. There are dressings that can facilitate autolytic debridement; the retention of moisture at the wound bed can help to soften and liquefy the accumulated dead cells and fibrinous deposits. Therefore, the selection of an ideal dressing is determined by the presence of necrotic tissue and biofilms as well as the type of tissue and its coloration, healing time, frequency of dressing changes, nursing costs, and need of secondary dressings, antibiotics, or analgesics [7,41,46,47]. 

**Figure 1:**
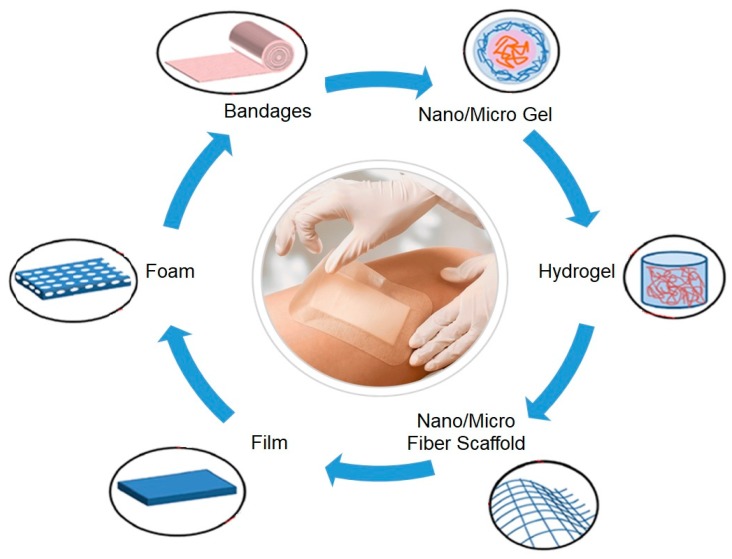
Structure of different wound dressings (adapted from [11], with permission from Elsevier, 2020).

The most widely used dressings in chronic wounds are the interactive/bioactive dressings, such as films, foams, hydrogels, hydrocolloids, and alginates (Figure 1) [48,49]. The films are flexible semipermeable dressings, impermeable to fluids and bacteria, and permeable to air and water vapor [50]. Hydrogels stand out by their insoluble, highly absorbent three-dimensional (3D) polymeric network, capable of maintaining a moist microenvironment at the wound bed. Hydrogels can be formulated as particles, sponges, films, and other 3D structures, and their porosity can be controlled by embedding particles of various sizes. These are particularly effective in wounds with minimal to moderate exudates [51,52,53,54]. Hydrocolloids typically consist in carboxymethylcellulose, pectin, or gelatin. In their intact state, hydrocolloids are impermeable to water vapors, but as the gelling process takes place the dressing becomes progressively more permeable. The loss of water enhances the ability of the dressing to cope with the exudates production and lower the pH, this way hindering the bacterial growth and contributing to an optimal, stable temperature, and moisture level that stimulates all phases of healing [46]. Alginates are usually classified as bioactive dressings, being available in the form of non-woven sheets and ropes or as calcium-enriched fibrous structures that are capable of absorbing fluid up to 20 times their weight [55,56]. Upon contact with the wound, calcium is exchanged with the sodium from the exudates turning the dressing into a gel. Because of this exchange, alginates act as a hemostat and are, therefore, useful in managing bleeding wounds. They also activate human macrophages to produce tumor necrosis factor-α (TNFα), which initiates the inflammatory signals. However, these dressings might not be the most suitable to fight infections, as they generate an environment that is conducive to bacteria proliferation [50,55,56]. 

As seen in the alginates, bioactive dressings can directly deliver active compounds to the wound. They may also be composed of materials with endogenous activity which play an active role in the healing process, by activating or driving cellular responses [57,58]. Various antibiotics, vitamins, proteins, minerals, enzymes, insulin, growth factors, cells, and antimicrobial agents have been used in this class of dressings to accelerate healing [26,29,59]. 

The production of nanofibrous dressings reinforced with active biomolecules has been accomplished through various techniques, including electrospinning, melt-blowing, phase separation, self-assembly, and template synthesis [45,60]. The electrospinning technique is perhaps the most researched as it allows the production of porous, randomly-orientated structures that mimic the 3D architecture of collagen fibers that are found within the ECM of normal skin [61]. This technique might be used to produce dressings belonging to each of the former categories (films, hydrogels, hydrocolloids or alginates) in its entirety or partially. Through simple blend with the polymer solution, while using one nozzle or multiaxial nozzles (coaxial or triaxial nozzle), core/shell, smooth and continuous structures may be engineered to increase the efficiency of the incorporated biomolecules and control their release kinetics. The incorporation of biomolecules within the polymeric solutions affects their viscosity and conductivity, which play a major role in their electrospinnability and in the resultant nanofiber morphologies. Alternatively, surface functionalization post-electrospinning, via chemical or physical methods, has been proposed. Electrospun nanofibers that are incorporated or functionalized with antimicrobial agents have shown enhanced antibacterial performance compared to traditional dressings. Depending on the application, the addition of specialized biomolecules to these nanofibrous networks may serve as platforms to increase oxygen exchange and absorption of exudates, and/or to stimulate proliferation, migration, and differentiation of cells, while promoting nutrient supply and controlling fluid loss [22,62,63,64,65].

Cellulose being a natural polymer has attracted lots of attention for biomedical applications, due to its inherent features, such as biodegradability, low price, abundance, renewability, high mechanical strength, and lightness. Considerable research has been undertaken on the use of cellulose, cellulose derivatives and nanocellulose for the production of electrospun 3D nanocomposites [66]. The most widely researched and employed cellulose derivative is CA, the acetate ester of cellulose. CA electrospun fibers have shown excellent biocompatibility and biodegradability, good thermal stability and chemical resistance [67]. For biomedical applications, the surface and structure modifications of CA-containing nanocomposites are commonly done. For instance, to generate a CA nanofiber mat with a honeycomb-like structure, F. Hamano et al. combined the electrospinning technique with a very simple oil spray method [68]. K.I. Lukanina et al., on its turn, generated a sponge-nonwoven CA matrix by filling the electrospun mat with chitosan and collagen and posteriorly freezing and vacuum drying the combination [69]. The natural, microbial, and biodegradable thermoplastic polymer poly(hydroxybutyrate) (PHB) was blended with CA and the processed by electrospinning to generate fibrous nanocomposites with a considerable capacity to induce cell adhesion and proliferation. At a higher CA content, it was seen that amorphous regions were more common and that the loss of fiber integrity occurred more quickly [70].

Recently, nanocelluloses have gained more interest in the biomedical field, due to their unique properties of low cost, biodegradability, biocompatibility, low cytotoxicity, outstanding mechanical properties, availability, and sustainability [71]. The great amount of -OH groups on the surface of these nanomaterials favors the formation of hydrogen bonds, playing an important role in promoting the adhesion between nanocellulose and other polymeric materials within the nanocomposites, aside from enhancing their water retention capacity [72]. Among the many types of nanocellulose available, bacterial cellulose (BC) has already been successfully applied, starting its commercialization in wound dressings in 1980 by *Johnson & Johnson* (New Brunswick, USA). A Brazilian company, *BioFill Produtos Biotecnologicos*, already created a new wound healing system that was based on BC, and *Lohmann & Rauscher*, a German company, has commercialize the Suprasorb X®. Bioprocess® and XCell® are other wound dressings that are also in the market. BC is capable of maintaining a moist environment at the wound bed and to absorb exudates during the acute inflammatory phase [73]. 

Many studies have been conducted to engineer bioactive dressings capable of facing the rising of microbial resistance pathogens without compromising the healing process. In the following sections, a complete review and discussion of the most successful alternatives, containing cellulose and its derivatives, to the conventionally used dressings was provided. Special attention will be given to clean strategies and to the issues still faced in this line of research, as the environment conservation remains a challenge and a major focus of this work. 

## 3. Cellulose and Its Derivatives

### 3.1. Cellulose

Cellulose is the most abundant organic, eco-friendly polymer on Earth. It is a polysaccharide that consists of D-glucopyranose units (commonly composed by 10,000–15,000 units, depending on the source) that are linked by a covalent β-1,4-glycosidic bond through acetal functions, between -OH groups of C4 and C1 carbon atoms, that forms a linear and high molecular weight homopolymer [74,75]. For each anhydroglucose unit, the reactivity of the -OH groups on different positions is heterogeneous. The -OH at the 6th position acts as a primary alcohol, whereas the -OH in the second and third positions behave as secondary alcohols. It has been reported that, on the structure of cellulose, the -OH group at the sixth position can react ten times faster than other -OH groups, while the reactivity of the -OH on the second position was found twice as high of that of the third position [71]. The many -OH groups that are present in the cellulose backbone establish numerous intra- and intermolecular bonds that result in its semicrystalline structure. However, this molecular structure may undergo modifications, depending on the source of the material, method of extraction, and treatment, giving rise to different polymorphs. There are four types of polymorphs of crystalline cellulose (I, II, III, IV). Cellulose I, which is also known as “natural” cellulose, is sourced in its vast majority from plants, tunicates, algae and bacteria, being a structural component in cell walls. Because its structure is thermodynamically metastable, cellulose I can be converted into cellulose II or III. Cellulose II is the most stable structure and it can be produced either by regeneration (solubilization and recrystallization) or by mercerization (aqueous sodium hydroxide treatments). This type of cellulose has a monoclinic structure and it has been used in the production of cellophane, Rayon, and Tencel. Cellulose III results from subjecting cellulose I and II to alkaline treatments, while cellulose IV originates from the thermal treatment of cellulose III [76,77]. Another important parameter that is influenced by the source and processing of cellulose is the degree of polymerization (DP), which is the number of monomer units in the polymer backbone and affects the material viscosity and mechanical properties. For instance, while cellulose from wood pulp has only 300–1,700 units, BC has a DP of 800–10,000 repeat units [78]. 

The primary natural source of cellulose is the lignocellulosic material that is present in wood (40–50 wt.%). It can also be extracted from vegetable fibers like cotton (87–90 wt.%), jute (60–65 wt.%), flax (70–80 wt.%), ramie (70–75 wt.%), sisal, and hemp. However, most of these sources require large arable spaces and considerable amounts of fresh water, fertilizers, and pesticides. Additionally, cellulose can also be produced from bacteria, algae, fungi, and some animals (e.g., tunicate) [74]. To reduce the environment impact associated with its production, strategies are being developed to effectively reuse wood pulp and agricultural and food wastes, or even take advantage of wastes from the textile industry (e.g., used garments) to recover cellulose and produce new fibers with similar properties to those regenerated from conventional wood pulp [74,78,79]. Table 1 introduces some of the alternative sources of cellulose and their inherent pretreatments to obtain an efficient extraction and new unconventional solvents systems that are applied in their solubilization. For instance, the solvent *N*-methylmorpholine-*N*-oxide (NMMO), which is used in Lyocell production (rayon fiber obtained from wood pulp), has been successful in dissolving cellulose, however it entails high costs and high temperatures; the NaOH/thiourea solvent system has been used in green processes for the production of regenerated cellulose textile fibers, yet it also entails limitations, namely the inability to effectively dissolve high degree polymerized cellulose or high concentrated cellulose solutions. The ionic liquids (IL) are another alternative that has attracted lots of attention for their effectiveness. However, even though they are compatible with cellulose, their elevated cost, toxicity and incapacity to be reused has hindered large-scale, multi-filament productions [80]. 

Cellulose has attracted considerable interest in the last years because of its potential for generating several high-value products with low impact on the environment and low cost [81]. Among its important characteristics for large-scale production, the cellulose intrinsic mechanical, chemical, and biological properties make this polymer extremely suitable for applications in composite engineering, food science, filtration processes, paper engineering, and medical engineering [82]. The clinical application of cellulose-containing 3D scaffolds includes repair, reconstruction, and regeneration of almost all types of tissues in the mammalian organism; this polymer endows scaffolds with the ability to support cell adhesion and growth. In wound dressings, it is no accident that these cellulose-based materials have been used since the mid-1970s, in the form of cotton gauze or non-woven mixtures of rayon and polyester or cotton fibers, since they are capable of absorbing excess exudates through their bulk polar groups (-OH) and allow for the production of highly porous structures, permeable to air, steam, and heat, ensuring the patients comfort [83]. 

Nowadays, with the rise of nanotechnology approaches, cellulose nanofibers have been engineered by electrospinning in the form of nanocomposite wound dressings that not only protect the wounds, but they are capable of releasing drugs that inhibit post-operative adhesions, stimulate hemodialysis and hemostasis, and repair tissue defects [20]. Cellulose electrospun nanofibers have been studied due to their ultrafine and highly porous structure, biocompatibility, biodegradability, hydrophilicity, low density, thermostability, low thermal expansion, and easy chemical modification [20,84]. Even though research continues in this field, the disappointing mechanical properties and the difficulties in processing cellulose in the form of nanofibers via electrospinning remain very important challenges. Its strong inter- and intra-molecular interactions originated from hydrogen bonding, and its rigid backbone structure is responsible for its insolubility in most conventional solvent systems and its inability to melt [85]. Several strategies have been proposed to address these limitations. For instance, cellulose nanofibers have been obtained via direct electrospinning by using NMMO (one of the most popular cellulose solvents), lithium chloride/dimethylacetamide (LiCl/DMAc), or ionic solvents,nsuch as 1-ethyl-3 methylimidazolium acetate 1-ethyl-3 methylimidazolium acetate (EmimAc) [86]; however, these processes are cumbersome and very expensive. This has limited the use of cellulose in nanofibrous constructs for wound healing and has increased the use of cellulose derivatives, particularly CA [87].

### 3.2. Cellulose Acetate (CA)

Many cellulose derivatives have arisen in order to overcome the limited solubility of cellulose in general organic solvents [28]. CA is one of the most important cellulose derivatives, with applications in textile, plastics, cigarette filters, diapers, sensors, LCD screens, catalysts, coatings, semi-permeable membranes for separation processes, nano and macro composites, and fibers and films for biomedical devices [97,98]. This polymer is under great consideration in the biomedical industry due to its biodegradability, biocompatibility, mechanical performance, non-toxicity, high affinity, good hydrolytic stability, relative low cost, and excellent chemical resistance [99]. These exceptional properties have driven the processing of CA-containing polymeric blends in the form of electrospun nanofibrous composites, in this way generating a new smart option for biotechnology and tissue engineering, drug delivery systems and wound dressing applications [25,26,27]. Electrospun CA has also been used to immobilize bioactive substances as vitamins and enzymes, biosensors, bio-separation, and affinity purification membranes, while non-porous CA have been used for stent coatings or skin protection after burns or wounds. Interestingly, CA has also been proven as an effective material for tissue scaffold engineering, providing good mechanical stability, and ability to mimic the extracellular matrix for cell attachment, growth, and advanced formation of targeted tissues (e.g., bones and skin) [100]. Chainoglou et al. has even demonstrated the possibilities of CA for heart valve tissue engineering, through a successful promotion of cardiac cell growth and proliferation [101]. Each one of these applications is dependent on their overall properties, which, in turn, are dependent on the polymer chemical characteristics, such as molar mass, molar mass distribution, DP, and degree of substitution (DS) [102]. 

DS can be easily understood as the average number of acetyl groups replacing hydroxyl groups per glucose unit. The maximum degree of acetylation is obtained when all of the -OH groups are replaced by acetyl groups, which leads to a DS that is equal to three [103]. DS is a parameter that demands a detailed understanding as it affects the chemical, physical, mechanical and morphological properties of the polymer, altering its polarity, aggregation behavior, biodegradability, and solubility [103,104]. 

The acetylation process reduces CA crystallinity and insolubility in water [100]. Industrially, CA is produced by the reaction of cellulose with an excess of acetic anhydride in the presence of sulfuric acid or perchloric acid as catalysts, in a two-step process of acetylation, followed by hydrolysis [105,106]. These steps are taken due to the heterogeneous nature of the reaction, since the structure of cellulose is made up of amorphous parts, which react first, and crystalline parts, which then react second; hence, being impossible to synthesize directly partially substituted CAs. An extra hydroxylation step is required for producing CA with the desired DS [21]. CA production entails very high-quality cellulose as raw material, with a high alpha cellulose content [21,105,106,107,108,109,110]. This high-quality cellulose is generally obtained from cotton or wood dissolving pulp, where the cellulose rate is generally more than 95%. However, this is considered to be an expensive material. Alternative sources of cellulose have been researched, finding lignocellulosic biomass as an attractive alternative due to its renewability and large availability worldwide. Preliminary work has uncovered some important sources of CA that are based on biomass that include microcrystalline cellulose (MCC), cotton linter pulp, wheat straw pulp, bamboo pulp, bleached softwood sulfite dissolving pulp, bleached hardwood kraft pulp (HP) [21], oil palm empty fruit bunches [105], sugarcane straw [106], waste cotton fabrics [107], sugarcane bagasse [108], sorghum straw [109], babassu coconut shells [110] and waste polyester/cotton blended fabrics (WBFs) [111]. Still, there is a long way until process optimization occurs, since there are major barriers to the production of cellulose-containing products from agricultural residues, including the heterogeneity of the raw material, the processing conditions reproducibility, the heterogeneous phase of the synthesis reaction, the difficulty of purification, the effluent disposal, and the control of product quality [112]. 

Many researchers apply alkali or acid pretreatment to remove lignin and hemicellulose of material resources to increase the yield of CA production from wastes, affecting the cellulose crystalline structure, which then becomes more amorphous [110,112,113]. For instance, L. Cao et al. used diluted phosphoric acid at different temperatures and B. Ass et al. used NaOH to disrupt the crystalline structure of cellulose, which increases the amorphous region and renders cellulose more accessible to acetic anhydride, resulting in an acetylation process more effective for CA production [113,114]. Additionally, H.R. Amaral et al. resorted to acid pretreatment of babassu coconut shells to increase the yield of the acetylation of cellulose to obtain CA [110]. These works have shown the importance of pretreating lignocellulosic biomass to increase CA synthesis yield. However, bio-residues from these treatments are a serious environmental challenge due to their aggregation with household wastes, causing disturbances in the ecological cycle of the soil, and resulting in soil infertility and environmental pollution; thus, attention should be urgently paid [110,113,115]. A great opportunity has arisen to explore more of these bio-based polymers and their alternative production methods, given the current environmental and energy policies. Table 2 offers a general overview of this topic, covering some of the most effective alternative solutions for CA processing. 

### 3.3. Nanocellulose

The increased demand for high-performance materials with tailored mechanical and physical properties has elevated the nanocellulose status to one of the most attractive renewable materials for advanced medical applications. Nanocellulose is a considered to be a new generation of nanomaterials that combines important cellulose properties, including high specific strength, hydrophilicity, low density, flexibility and chemical inertness, with the ability to be chemically modified to incorporate specific features at the nanoscale [120,121]. In biomedicine, its exceptional water-retention capacity and large surface area that are associated with enhanced cell attachment, proliferation, and migration with no reports of toxic responses, has increased its desirability for a variety of uses that include packages, membranes for hemodialysis, vascular grafts, drug delivery systems, wound dressings, and tissue engineering strategies [122,123,124].

**Figure 2:**
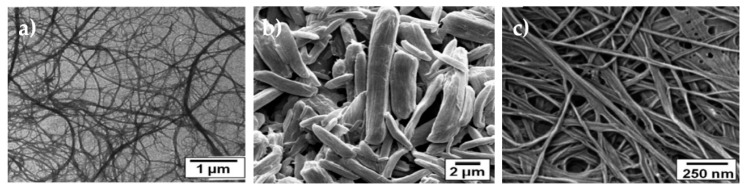
**a**) TEM image of cellulose nanofibers (CNFs); **b**) SEM image of cellulose nanowhiskers (CNCs) that has been deagglomerated; and, **c**) SEM image of BC (adapted from [77] with permission from Royal Society of Chemistry, 2020).

Nanocelluloses can be classified in three main categories: (1) cellulose nanofibers (CNFs), also known as microfibrillated cellulose (MFC), and nanofibrillated cellulose (NFC); (2) cellulose nanocrystals (CNCs), also designated by nanocrystalline cellulose (NCC) or cellulose nanowhiskers (CNWs); and, (3) BC or also named bacterial nanocellulose (BNC) [121]. The major difference between the CNFs and CNCs lies in their dimensions and crystalline structure. While CNFs have lengths in the microscale and diameters in the nanoscale, CNCs have both length and diameter that are in the nanoscale [125]; more precisely, CNFs are fibrils with lengths of a few micrometers and with diameters that range between 3 and 50 nm, whereas CNCs have a rod-like nanocrystal configuration with lengths ranging from 10 to 500 nm and diameters of few nanometers (Figure 2) [120]. Differences in the CNFs and CNCs crystalline structure result from their extraction process. CNF contains either crystalline regions or amorphous regions, in which the amorphous domains provide a certain flexibility to NFC. In turn, CNC are mostly nanoparticles that are made predominately of pure crystalline cellulose [126,127]. 

The isolation of CNFs from different cellulosic origins is accomplished by means of mechanical treatments, often in combination with some chemical or enzymatic pretreatment followed by a disintegration step. The most common chemical pretreatments are perhaps those that render the pulp fibers (used when the source is wood) charged, e.g., anionic or cationic. This modification increases the electrostatic repulsion between the fibers, which is beneficial in the subsequent mechanical treatment steps, as it further promotes the fibers disintegration into nanofibers. The most used mechanical processes are the high-pressure homogenization, microfluidization, refining, and grinding, while the least used are the electrospinning, ultrasonication, cryocrushing, and steam explosion. However, all of these mechanical processes demand high energy consumption. Hence, chemical and enzymatic pretreatments, such as cationization, hydrolysis, (2,2,6,6-Tetramethylpiperidin-1-yl) oxyl(TEMPO)-mediated oxidation, acetylation, and silylation, have been used to ease the mechanical treatment and, thus, reduce the energy consumption, while attaining a desirable surface chemistry. Still, caution should be taken during mechanical processing, since the nanofibers length depends on the degree to which the material has been exposed to this processing step. In addition, the cellulose source will also play a major role in the final product, as it determines the pretreatments that are to be carried out [127,128].

Two steps are also required to process CNC from raw cellulose: (1) homogenization pretreatment/purification, and (2) the separation of the purified cellulose into nanocrystals. To obtain cellulose nanocrystals, cellulose can be directly hydrolyzed. Acid hydrolysis has been the method of choice for many years to produce CNC. Generally, it requires sulfuric and hydrochloric acids, which starts by dissolving the disordered or para-crystalline regions, leaving behind the crystalline domains or the CNC that possess a higher acidic resistance. The temperature and time of hydrolysis, nature, and concentration of the acids and the fiber-to-acid ratio play an important role in the CNCs particle size, morphology, crystallinity, thermal stability, and mechanical properties. It is worth noting that surface sulfate esters are introduced to the CNCs during sulfuric acid hydrolysis, conferring the surface with a highly negative charge and making it accessible, for instance, to enzymes or proteins, a desirable outcome in biomedical applications [71]. Aside from hydrolysis, other methods have been reported to isolate CNCs, such as enzymatic hydrolysis, mechanical refining, ionic liquid treatment, subcritical water hydrolysis, and oxidation processes. Different sources, like plant cell walls, cotton, microcrystalline cellulose, algae, animals, and bacteria, have been used to obtain CNCs [81,129]. Like CNFs, the geometric dimensions and the final properties of the CNCs are directly dependent on the cellulosic source, the post- or pretreatments, and the subsequent preparation and processing conditions [130]. CNCs are characterized by their biocompatibility, biodegradability, high level of crystallinity (54–88%), excellent stability and mechanical performance (high strength as well as modulus), exceptional optical properties, and flexible surface chemistry [123,131].

BC is another class of nanocellulose materials that has been engineered with the goal of surpassing the limitations of cellulose and other natural or synthetic materials [132]. BC is chemically similar to the cellulose obtained from plants; however, it is free from lignin, pectin, and hemicelluloses, and it has a very low amount of carbonyl and carboxyl in its structure. BC is a biocompatible, highly porous, and highly crystalline (84–89%) polymer, with a high degree of polymerization (up to 8000), a finer web like network, and an extraordinary mechanical strength, particularly in the wet state, which was comparable to other nanofibers from plants. It is characterized by a superior water-retention capacity, and the ability to accelerate granulation tissue formation, making it very attractive for wound healing (Figure 2) [75,126]. Another, relevant property of BC is its in situ moldability (e.g., shaping during biosynthesis) [133]. Many bacteria from the genus *Acetobacter, Agrobacterium, Pseudomonas, Rhizobium, Achromobacter, Bacillus, Azotobacter, Enterobacter, Klebsiella, Salmonella*, and *Sarcina* have been reported to secret BC as a protection mechanism against ultraviolet light or other microorganisms, like fungi and yeasts [126,129]. BC properties are highly influenced by the origin organism and culture conditions [128]. The most common BC producer is the gram-negative bacteria *Gluconacetobacter xylinus*, previously known as *Acetobacter xylinum* and secretes cellulose during metabolism of carbohydrates [132]. The fermentation method that is used more to produce BC has been the static culture, which increases the yields of BC, by producing BC layers of several centimeters of thickness under the surface of the culture medium. Here, however, it is necessary to monitor the media pH, since the accumulation of acids, such as gluconic, acetic or lactic, decreases the pH far below the optimum for bacteria growth and cellulose production. Alternatively, agitated cultures, airlift bioreactors, rotating disk bioreactors, stirred tank reactors with a spin filter, biofilm reactors with plastic composite supports, and trickling bed reactors may also be employed in the production of this cellulose type, preventing the conversion of cellulose-producing strains into cellulose-negative mutants [128]. BC can be produced in various forms, depending on the fermentation method; pellicles arise under static culture condition, while fibrils and sphere-like particles emerge under motion conditions [134]. The wastes from several industries (often rich in sugars), e.g., domestic, agricultural, cotton-based textiles, among others, are also gaining significance as carbon sources for BC production, as evidenced in Table 3 [132]. Celluloses with different degrees of crystallinity can be produced, depending on the source and culture production [133]. This is one of the most important properties in BC, since the crystalline microfibrils in its structure are responsible for its high tensile strength (200–300 MPa) and thermal stability. Its poor solubility in physiological media, as well as the absence of cellulases and beta-glucanases, which increase the stability and functionality of the polymer, has increased the interest of BC as additive or base for potential new biomaterials. To date, BC has been employed in the development of biomaterials for wound dressings, blood vessels, dental implants, scaffolds for tissue engineering of cornea, heart valve, bone and cartilage, and drug delivery applications [134]. 

## 4. Application in Wound Healing: Synergistic Effect with Specialized Biomolecules

In wound care, infections are a major concern, since they delay the healing process, leading to tissue disfigurement or even patient death. *S. aureus* and *P. aeruginosa* are the most common bacteria that are isolated from chronic wounds, being *S. aureus* usually detected on top of the wound and *P. aeruginosa* in the deepest regions. They can express virulence factors and surface proteins that affect wound healing. The co-infection of *S. aureus* and *P. aeruginosa* is even more problematic, since the virulence is increased; both bacteria have intrinsic and acquired antibiotic resistance, making the clinical management of these infections a real challenge [147]. In fact, the World Health Organization considers *P. aeruginosa* as one of the organisms in urgent need for novel, highly effective antibacterial strategies that combat its prevalence. Multiple strains of *S. aureus*, including methicillin-resistant and vancomycin-resistant strains, have been identified as high priority microbes in the fight against antimicrobial resistance build up [15]. In addition to the above, other microorganisms, such as beta-hemolytic streptococci, and mixtures of Gram-negative species, such as *E. coli* and *Klebsiella* strains, are also present in wounds. Bacterium native to human skin such as *Staphylococcus epidermidis* (Gram-positive), may also turn pathogenic when exposed to systemic circulation in the wound bed [148]. Therefore, immediate care of open wounds is pivotal in preventing infection [149]. To treat this problem, new alternatives of wound dressings have emerged with incorporated bio actives that are capable of fighting these infections and accelerating the healing process.

The performance of bioactive dressings processed via electrospinning is dependent on the polymer or polymer blends properties (i.e. hydrophilicity and hydrophobicity), drug solubility, drug-polymer synergy, and mat structure. Antimicrobial agent-loaded electrospun mats have shown superior performance to films produced by other techniques, in regard to water uptake (four to five times superior), water permeability, drug release rate, and antibacterial activity [9].

Drugs, nanoparticles, and natural extracts (Table 4) are some of the antimicrobial agents that have been incorporated in nanofibrous dressings, in order to reduce the risk of infection [61]. These compounds have been used for their anti-inflammatory, pain-relieving, vasodilation, and antimicrobial features [11].

Several researchers claim that producing cellulose-based electrospun mats is a big challenge due to its highly crystalline structure, long chain length, increased rigidity, and strong inter- and intramolecular hydrogen bonding [150]. Selecting a proper solvent, adding other complementary polymers, or converting cellulose into its derivatives can facilitate this task. As seen in Section 3.1, the solvents or solvent systems most used for cellulose are the ILs, aqueous alkali/solvents (NaOH/urea), and polar aprotic solvents in combination with electrolytes (DMAc/LiCl); however, these are not very volatile, not being completely removed during electrospinning and, thus, limiting the use of cellulose in large scale productions. A proper solvent system is also very important in attaining appropriate viscosity levels, required for a successful electrospinning process. In fact, this is such an important processing parameter that to guarantee proper polymer solubilization, heaters have been placed within the electrospinning apparatus generating a new system, the melt-electrospinning (minimize the viscosity of spinning dopes) [151]. The option of transforming cellulose into its derivatives, such as CA, cellulose acetate phthalate (CAP), *ethyl cellulose* (EC), carboxymethyl cellulose (CMC), hydroxypropylcellulose (*HPC*), among others, is by far the most recurrent alternative to reduce the complexity of processing cellulose via electrospinning. Besides, most of these derivatives require different pHs for solubilization, which is a great advantage in biomedical applications [152]. 

Modifications have been proposed to increase the effectiveness of immobilized drugs, natural compounds, peptides, or other biomolecules within a cellulose-based nanostructured surface. For example, Nada et al. activated CA by introducing azide functional groups on the residual -OH groups of the polymeric chains, enhancing the release kinetics of capsaicin and sodium diclofenac from the electrospun mat and, thus, promoting patient relief [153]. To confer biocidal properties to CA nanofibers, Jiang et al. modified their surface with 4,4’-diphenylmethane diisocyanate (MDI). This resulted in a 100% inactivation of *S. aureus* and a 95% of *E. coli* within 10 min of exposure, and complete death after a 30 min contact [154]. Nano complexes with CNCs were developed with cationic b-cyclodextrin (CD) containing curcumin by ionic association and used in the treatment of colon and prostate cancers [155]. Nanocellulose has also contributed to the development of new and more efficient strategies for these biomolecules’ delivery. The three -OH groups that were present in each individual glucose unit originate a highly reactive structure, which allows interaction with other molecules or with enzymes and/or proteins, contributing to overcome the low solubility of most drugs in aqueous medium [127]. Besides, the -OH groups can also be tailored by physical adsorption, surface graft polymerization, and covalent bonding to further improve the performance of the biomolecules. As a consequence of the bonds established, strong polymer-filler interactions are generated, significantly increasing the mechanical properties of material [156]. Nonetheless, the in vivo behavior of nanocelluloses is still little explored. Studies have reported that its toxicity depends on the solution concentration and its surface charges. In recent literature, nanocelluloses have not shown any toxicity at concentrations lower than 1 mg/mL; however, there are studies that reveal a concentration-dependent apoptotic toxicity of CNFs at 2–5 mg/mL. Additionally, anionic nanocelluloses, e.g., carboxymethylated CNF, have been reported to be more cytotoxic than cationic nanocelluloses, e.g., trimethylammonium-CNF [34]. Toxicity effects might arise from the diversity of chemical structures and properties between cellulose types and sources. Among nanocelluloses, BC is considered to be the most biocompatible and has already been applied in wound dressings [71]. Still, its electrospinnability is very challenging for the same structural reasons of cellulose [150]. 

The incorporation of BC into synthetic and natural polymers has been carried out to enhance their morphological features as well as physicochemical and biological performances. A wide variety of polymers, such as chitosan, polycaprolactone (PCL), polyethylene oxide (PEO), ethylene vinyl alcohol (EVOH), polyvinyl alcohol (PVA), polylactic acid (PLA), polyacrylonitrile (PAN), polyester, silk, and zein, have been blended with BC and processed by electrospinning. Functionalization with 3-aminopropyl triethoxysilane (APS) has been attempted to further enhance cell attachment and antibacterial properties of BC-containing electrospun membranes for wound healing. BC membranes grafted with two organosilanes and acetyled have also shown an improved moisture resistance and hydrophobicity [134]. Naeem et. al even synthetized in situ BC on CA-based electrospun mats in a process known by self-assembly to produce a new generation of wound dressings [157]. 

Even though CNF has already been applied as a reinforcing agent in many polymeric composites via electrospinning, no reports have been found regarding the incorporation of biomolecules along its fibers [158]. As such, in the following sections BC and CNCs will be explored in more detail.

### 4.1. Drug Loading

Numerous hydrophilic and hydrophobic drugs have been incorporated into electrospun polymeric nanofibers. In general, the polymer is dissolved in an organic solvent and the drug is slowly added to the polymer solution under stirring in order to guarantee a homogeneous distribution. This strategy allows for a large amount of drugs to be loaded into the nanofibers by simply adjusting the final concentration of the solution. However, adding drugs directly to a polymer solution alters its conductivity, viscosity, and surface tension, affecting the electrospinnability of the polymer and the morphology of the obtained nanofibers. Besides, in this scenario, drugs tend to very rapidly leach in an aqueous environment [193], since they are preferentially located at or near the fibers’ surface [166]. The conventional electrospinning technique allows a somewhat control of drug release by modulating the pores size and density, and the polymers degradation rate; still, bursts of drug followed by cytotoxic effects remain [63]. Several research teams have focused on developing new drug delivery systems with a so-called effective controlled release to overcome this weakness. Multiple-fluid, coaxial and triaxial electrospinning approaches, capable of generating complex nanostructures, may allow a more effective control of this initial burst release by confining part of the drug to the fiber core and another to the surface. This way, during dissolution, the molecules at the core need to diffuse through an insoluble shell until reaching the bulk solution [169]. However, this is not always as straightforward. Yu et al. compared nanofibers that were produced from coaxial electrospinning at varying feeding rates. They realized that by varying just this one parameter the morphologies of the fibers obtained were completely different and that to condition drug release. They proved that the production of high quality ketoprofen-loaded CA nanofibers is not simply a result of dilution of the core solution by the sheath solvent. The most uniform fibers, with the smallest diameters, and extended drug release time were those that were produced with the lowest feed rate being applied to the sheath of CA [171]. 

**Figure 3:**
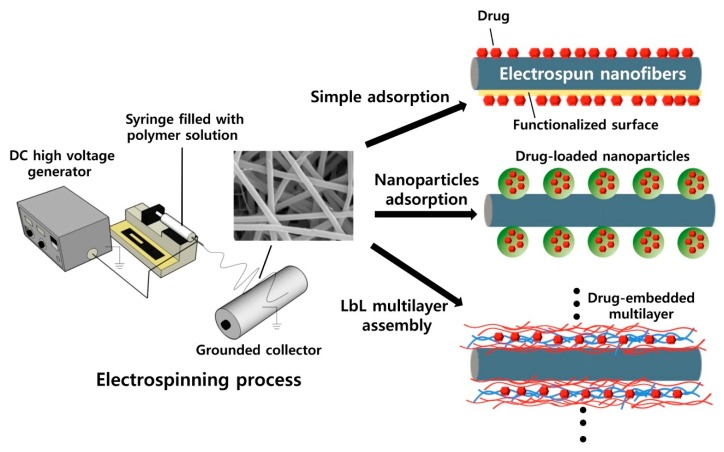
Three modes of physical drug loading on the surface of electrospun nanofibers (reproduced from [194], with permission from Elsevier 2020).

Many attempts have been made to optimize the release kinetics of drugs over time resorting to different immobilization methods (Figure 3). Table 5 compiles some of the most successful formulations of drug and electrospun nanocomposites containing cellulose, CA, or any variation of nanocellulose. As explained earlier, CA is the oldest, most researched derivative of cellulose, and, as such, the drug loading of CA-containing electrospun wound dressings are more recurrent.

### 4.2. Nanoparticles (NPs)

Nanotechnology tools, particularly NPs, have been recognized as occupying a fundamental role in promoting wound healing, with reports on their exceptional antimicrobial, angiogenesis, immunomodulation, and cell and drug delivery, leading the way to new strategies for improving the response to antimicrobial and tissue regeneration therapies [8]. 

NPs are classified in light of their impact in cellular uptake, dimension (1–100 nm), shape, role, and nature (inorganic and organic). Carbon-based, metal and metal oxide, semiconducting and ceramic NPs are classified as inorganic, while organic NPs integrate those that are produced from polymers and derived from biomolecules [195]. NPs can act as delivery vehicles, protecting and releasing active compounds locally, or by intervening in specific functions via their intrinsic properties [6]. Their antibacterial potential results from their production of reactive oxygen species and their capability to bind and disrupt DNA or RNA functions that obstruct microbial reproduction [130]. By associating NPs with a textile or polymeric matrix synergistic actions can be revealed, generating a new formulation of active dressings [6,148]. The NPs with antimicrobial activity that have been explored in combination with dressings are the bioactive glass, gold, copper, cerium, zinc oxide, carbon-based, titanium dioxide, gallium, nitric oxide, and AgNPs [130]. These display bacteriostatic and bactericidal capacity, reduced in vivo toxicity (at low concentrations), are low cost, and possess physical, chemical, and biological features that trigger complex biological responses [196]. AgNPs can be highlighted from the group for their proved potential against multidrug resistant (MDR) bacteria [188]; they are capable of blocking the respiratory pathways of specific enzymes and damage the bacteria DNA, or even block the action of selected proteins involved in key metabolic processes [189,197]. In addition, AgNPs have been associated with decreased levels of pro-inflammatory cytokines TNF-α and IL-8 and increased levels of anti-inflammatory cytokine IL-4, EGF, KGF, and KGF-2, with enhanced fibroblast migration and differentiation into myofibroblasts, macrophage activation, and improved proliferation and relocation of keratinocytes, all being very important phenomena in wound healing [198,199]. AgNPs are already clinically used, being found in dressings, gels or ointments for topical treatment of infected burns and open wounds, including chronic ulcers [6]. However, there are still some adverse effects arising from the excess use of AgNPs. At high concentrations, AgNPs may be toxic to the human cells, by inhibiting the recruitment of immune cells, the regrowth of epidermal cells, and, ultimately, hindering wound healing. Besides, like antibiotics, prolonged treatment with metal ions may result in the emergence of resistant bacterial strains. A balance between cell exposure and action against microorganisms is, therefore, required to prevent such events. Table 6 summarizes some of the most recent systems for wound healing that combine NPs with electrospun mats containing cellulose or its derivatives, in the most successful way. 

Nowadays, the growing awareness of the NPs impact in the environment has led to the development of more eco-friendly approaches for inorganic NPs synthesis, which justifies new choices of solvents and reductive and stabilizing agents [200]. In fact, there are now approaches that resort to microbes, fungi, and vegetable, fruit, and plant extracts to produce metal and metal oxide NPs. There is still a long way until the optimization of such alternatives; however, it is already clear their economic and environmental potential over the current physical and chemical technologies [201,202]. 

### 4.3. Natural Extracts

Biomolecules that are derived from natural extracts are gaining more interest in biomedicine as alternatives to overcome the concerns associated with the resistance and toxicity of antibiotics and the overuse of silver-based compounds [206,207]. The use of plant extracts for the treatment of wounds and wound-related diseases is a very common practice. Thymol, asiaticoside, curcumin, zein, acid gallic, and gingerol are some examples of bioactive molecules used in combination with cellulose derivatives-containing electrospun nanocomposites. Their bioactive properties arise from alkaloids, phenolic, flavonoids, and terpenoids compounds, which are also endowed with immunomodulatory activities, which make these biomolecules capable of controlling the inflammatory response. Besides, these compounds are also responsible for these biomolecules antibacterial, insecticidal, antiviral, antifungal, and antioxidant properties. 

The use of plant extracts in medicine dates back hundreds of years. For instance, natural extracts derived from *Aloe vera* such as emodin (3,8-trihydroxy-6-methyl-anthraquinone), an antioxidant compound, have been frequently used in the treatment of burns. Neem (*Azadiracta indica*) extracts containing omega fatty acids also have numerous medical and cosmetic applications. Ginsenosides found in the plant genus Panax (*Ginseng*) are often used in traditional Chinese medicine and exhibit anticancer activity. Indeed, various plant extracts and active components, formulated as nanofibers or nanoparticles, are regaining interest for therapeutic purposes [208], because of their low cost, bioavailability, and superior efficacy, with limited side effects, over the more current and synthetic alternatives.

Essential oils (EOs), which are extracted from aromatic plants, have intrinsic antibacterial, antifungal and insecticidal properties. Moreover, EOs are widely available natural compounds with a low degree of toxicity. They can be easily and efficiently combined with polymeric matrices to generate nanocomposites with improved antimicrobial features [67,168]; these are mainly conferred by active molecules present in their composition, namely terpenes, terpenoids, and other aromatic and aliphatic compounds. The EOs, and their respective components, hydrophobic character promote the partition of the lipids that are present in the bacteria cell membrane, increasing their permeability and, consequently, leading to the membrane rupture and leakage of intracellular content, ultimately inducing cell death. Therefore, EOs loaded dressings may act as powerful tools to circumvent bacteria multi-drug resistance in infected wounds [209]. In fact, studies have already shown the improved synergistic effect of the oregano EO with CA-based nanofibers against *S. aureus*, *E. coli* and the yeast *Candida albicans* (*C. albicans*), as a result of the potent antimicrobial character of the oregano oil molecular components carvacol and thymol [67]. Cinnamon, lemongrass, and peppermint EOs that are loaded onto CA electrospun mats have shown similar outcomes. However, it was also seen that the morphology of the mat is a determinant factor in the EOs antimicrobial assessment as the nanostructure fibrous network developed might impair direct contact with large sized microorganism, such as *C. albicans*. Regarding the modified dressings cytotoxicity, even though fibroblasts and human keratinocytes could attach and spread on the fibers surface, cell viability seemed to decrease with exposure time. The anti-proliferative effect of EOs against eukaryotic cells has already been reported [168]. This is the greatest limitation to a large-scale use of EOs as antimicrobial and regenerative biomolecules in wound healing. Still, the capacity to design and engineer systems that allow a gradual and continuous release of EOs at concentrations below the cytotoxic, while using the electrospinning technique, has been improving and has already revealed very promising results. In fact, studies have shown that CA-based electrospun nanostructures loaded with EOs to display a higher capacity to retain water and aromatic compounds, thus reducing the initial drug burst and extending release over time, this way increasing the effectiveness of the therapy above other non-reticulated systems [179,180]. Table 7 presents some of the most recent EOs loaded electrospun systems containing cellulose, CA or nanocellulose formulations, in which the above-mentioned properties and outcomes are the most noticeable. Several of those works also report on the modifications introduced by the EOs to the fiber diameters and the relative porosity of the engineered mats, which intimately affect the cell proliferation, migration, and capacity of EOs release without an adverse biological response. 

### 4.4. Wound Healing Alternative Methods Containing Cellulose-Based Compounds

Wound healing is a highly complex process of tissue repair that relies on the synergistic effect of a number of different cells, cytokines, enzymes, and growth factors. A deregulation in this process can lead to the formation of a non-healing chronic wounds. Current treatment options are unable to meet the demand set by the environment surrounding these wounds. Therefore, multifaceted bioactive dressings have been developed to more efficiently respond to these wounds demands [206].

Surfaces have been physically and chemically modified, by changing the dressing topography or by introducing functional groups, like cell-recognizable ligands and bioactive molecules at the outermost layer, in order to improve the performance of electrospun polymeric nanofibers for skin regeneration. To accomplish such task, surface functionalization techniques, like the wet-chemical method, plasma treatment and graft polymerization have been applied. Pre- and post-electrospinning surface modifications are also very common; in pre-electrospinning bioactive molecules can be dissolved or dispersed in the polymeric solution, while in pos-eletrospinning physical adsorption, layer-by-layer (LbL) assembly and chemical immobilization are the most common strategies [61]. 

Alternatively to the earlier mentioned additives, drugs, nanoparticles, or natural extracts, other molecules, like growth factors, hormones, or enzymes, have also been incorporated onto nanofibrous dressings to promote wound healing [211]. Huang et al. produced CA nanofibrous mats that were used as a substrate to deposit LbL films, alternating between positively charged lysozyme-N-[(2-hydroxy-3-trimethyl-ammonium)propyl] chitosan chloride (LY–HTCC) compositions and negatively charged sodium alginate. The average fiber diameter increased with the increased number of bilayers, but only the samples that contained lysozymes were effective against bacteria [212]. Similar observations were made by Li et al. Here, lysozyme was combined with rectorite and electrosprayed onto negatively charged electrospun CA nanofibrous mats. The release profiles of lysozyme and its activity over time both demonstrated this formulation suitability for long-term applications [213]. Bio-based electrospun nanocomposites containing a pain reducing local anesthetic, the benzocaine (BZC), and the in situ pH-detecting dye bromocresol green (BCG) have been engineered to serve as a dual nano-carrier system for the treatment of infected wounds. BZC and BCG were introduced to CA-based nanofibers while using a single-step needleless electrospinning process. In vitro release studies demonstrated a pH dependent, controllable release of BZC, and confirmed the expected maximum drug release rate at pH 9.0, the average pH of an infected wound [214]. B. Ghorani et al. designed a β-Cyclodextrins (β-CD)/CA electrospun nanocomposite to efficiently trap and adsorb volatile molecules that are responsible for the unpleasant odors in chronic wounds. The data demonstrated an enhanced direct adsorption of a model odor compound, the hexanal (up to 80%), indicating the feasibility and potential of this formulation [24]. Vitamin A or retinol and Vitamin E or α-tocopherol have also been combined with CA solutions and electrospun in the form of cross-sectionally round, smooth fibers, with the average diameters ranging between 247 and 265 nm. The contents of Vitamin E and Vitamin A within the as-spun fiber mats were of ≈ 83% and ≈ 45%, respectively. Vitamin E was found to be more stable over time, with a maximum release of ≈ 95% of its loaded content after a 24 h period, against an ≈ 96% release of Vitamin A in only 6 h [215]. Cui et al., to improve the interaction between cells and scaffolds, modified the surface of thermoplastic polyurethane (TPU) nanofibers with CNF particles by ultrasonic-assisted technique and used polydopamine as binding agent. These composites increased cell attachment and viability, revealing excellent biological and mechanical properties [216]. In another approach Kolakovic et al. produced drug loaded CNF microparticles via the spray drying method and revealed a sustained drug release by means of a tight network that limited the drug diffusion from the system [217]. These studies offer new structures for the delivery of effective treatments in wound healing, in which the sustainability of the materials and the preservation of the environment are decisive factors during processing.

## 5. Conclusions and Future Perspectives 

Wound healing is a complex process that is regulated by three essential and distinct phases. Dysregulation or disruption of this process results in non-healing, very difficult to treat chronic wounds. In the last decades, remarkable progress has been achieved in the development of therapeutic approaches for these wounds. Electrospinning is regarded as one of the most effective tools for the production of dressings with a 3D structure that is similar to the skin extracellular matrix. These electrospun dressings display a large surface area-to-volume ratio and a porous structure that enhances homeostasis, exudates absorption, gas permeability, cell adhesion, migration, and proliferation and prevents the development of complicated infections. Herein, insights on the recent advances attained in the production of electrospun nanofiber meshes containing cellulose and its derivatives and modified with specialized biomolecules were provided. New, different, and more effective approaches have been developed for overcoming the concerns that are associated with the resistance generated by antibiotics and the overuse of silver compounds. Natural extracts from plants, alternative drugs, and organic and inorganic nanoparticles have been combined with selected nanofibrous systems based on cellulose components for an accelerated wound healing.

Even though many studies have reported on the availability of cellulose, its processing remains very challenging, with researchers turning to CA for facilitating dressing production via electrospinning. Indeed, CA is the most recurrent derivative of cellulose applied in wound dressings production, with many drug-loaded systems already engineered. Yet, nowadays, nanocellulose is gaining more ground by promoting binding with various biomolecules, including proteins and enzymes, via its highly available -OH groups that are also responsible for overcoming the low solubility of other forms of cellulose. These nanofillers have also contributed to significantly increasing the mechanical properties of wound dressings. Despite these advantages, more in vivo studies are required, since there is no consensus regarding its toxicity to human cells. From the collected data, it is clear that the different forms of cellulose presented are very attractive as renewable materials for wound dressings applications due to their high specific strength, high water-retention capacity, enhanced cell attachment, proliferation, and migration with no reports of toxic responses, and ability to be chemically modified to incorporate specific biomolecules. However, the incorporation of these agents is not always simple, with it being necessary to overcome the limitations that are associated with their electrospinnability. The correct selection of appropriated solvents, combination of polymers, pre-treatments to increase the solubility of these natural resources, and introduction of new chemical functional groups at the surface for biomolecule binding, are essential to obtain reproducible and effective wound dressings. The studies analyzed in this review reflect well the hard work around this subject and the increasing concern with the development of sustainable solutions that are still capable of accelerating the healing of wounds and preventing possible infections. There is still a long way for these formulations to reach large scale production with little environment impact. Even though, there are already greener alternatives resorting to clean solvents, low energy demand technologies, and biodegradable complementary polymers, there is still much work to be done to obtain a “green” and effective wound dressing to treat of infected wounds. 

## Figures and Tables

**Table 1:** Extraction of cellulose from different sources.

Raw material	Solvents	Methods	Observations	Ref.
Black 100% cotton jeans Blue 80/20% cotton/polyester jeans	DMSO	Pretreatment: (1) to dissolve the dyes, HNO_3_ (0.5–1.0 to 1.5–2.0 M) was used at 50 °C for 20 min. Cellulose recovery: (1) PES and other organic contaminants were dissolved in DMSO at 50 °C; (2) the bleaching process resorted to NaClO diluted in HCl for 2 h at 40 °C.	1.0 and 1.5 M HNO_3_ were sufficient to dissolve the dyes in 20 min; The complete dissolution of PES and other organic contaminants took 6 h for the blue and 10 h for the black samples; The solvents used were recovered as well as the extracted PES, turning the entire process highly sustainable.	[88]
Black 100% cotton samples Blue 80/20% cotton/polyester samples	HNO_3_ DMCHA	Pretreatment: (1) for dye removal various concentrations of HNO_3_ were applied to the samples at 50 °C; (2) to regenerate the acid from the solution, dyes were absorbed with activated carbon. Dissolution and extraction of PES: (1) pre-treated fabrics were exposed to various amounts of DMCHA at 50 °C to dissolve PES; (2) after, filtration was done with CO_2_ for 1 h to extract the solidified polymer and the solvent was regenerated. Recovery of cellulose: (1) the portion of cotton resultant from the PES dissolution was washed and dried.	1.0 M HNO_3_ applied for 15 min at 50 °C was sufficient for dye removal from the blue sample; To remove dye from the black sample HNO_3_ was used at 1.5 M for 20 min at 50 °C; 100% cotton samples required 10 h for PES and organic contaminants dissolution, while 80/20% cotton/PES needed 6 h; High purity cotton and PES fibers were recovered from the textile waste.	[89]
Post-consumer cotton waste: white and colored cotton wastes	Alkali/urea aqueous system: NaOH/ CH₄N₂O and LiOH/ CH₄N₂O;	Pretreatment: (1) cotton shirts were cut in small pieces; (2) these were hydrolyzed in H_2_SO_4_ and autoclaved at 120 °C for 12 min. Wet spinning: (1) dried hydrolyzed cotton was dissolved in two aqueous solutions, LiOH/Urea/dH_2_O and NaOH/urea/dH_2_O, at concentrations of 3.25% and 5%.	Uniform regenerated fibers were obtained with diameters ranging from 23.9 to 33.0 μm; A structural shift from cellulose I in the original/hydrolyzed cotton fibers to cellulose II in the regenerated fibers was observed; A small amount of dye was lost during hydrolysis but no dye leaching was observed during spinning; The intrinsic color of the regenerated fibers eliminates the need for dyeing processes.	[90]
Waste nylon/cotton blended fabrics (WNCFs)	[AMIM]Cl	Pretreatment: (1) WNCFs were subjected to cutting and shredding processes; (2) the pieces of WNCFs were dewaxed in Soxhlet apparatus with NaOH solution (2 wt.%) for 2 h at 80 °C; (3) dried WNCFs were immersed in boiling water for 2 h, and then dried again at 80 °C in a vacuum oven for 24 h. Cellulose recovery: (1) dried blended fabrics were mixed with IL, at 110 °C under stirring, until complete dissolution of cellulose; (2) the solution was filtered; (3) a cotton cellulose/[AMIM]Cl mixture was obtained; (4) the precipitate was washed with dH_2_O, and dried at 50 °C for 48 h.	[AMIM]Cl showed to be an effective solvent to extract cellulose from WNCFs;Optimal operation conditions were attained with 3 wt.% waste fabrics and 110 °C for 80 min; The crystal structure of cotton cellulose from WNCFs was transformed from cellulose I into cellulose II after separation from nylon 6 by [AMIM]Cl; The highest yield obtained from the regenerated cellulose films was of ≈ 58%.	[91]
Waste denim	[Bmim]OAc DMSO	Pretreatment: (1) samples were ground into powder; (2) to attain a DP of ≈ 1000, the powder was treated with 10% NaOH for various time periods; (3) the pretreated substrates were washed with dH_2_O until neutral pH was reached, and dried in an oven at 60 °C overnight; Cellulose recovery: (1) fibers are wet spun at a polymer concentration of 6 wt.% in the binary solvent system of [Bmim]OAc and DMSO at a ratio of 20/80; (2) filaments were extruded through the spinneret into a coagulation bath containing dH_2_O at RT; (3) the fibers were washed in warm dH_2_O (60 °C) for 2 h and air dried at RT.	The addition of an aprotic solvent (DMSO) accelerated dissolution of the cellulosic materials (pre-swelling) while reducing the viscosity of the spinning dope; Use of binary solvent system of IL and DMSO at high concentration (1/4) reduces the overall process cost; The regenerated discolored cellulose fibers had similar morphology and mechanical properties to those of viscose fibers.	[92]
Cotton waste garments (CWG)	NMMO	Pretreatment: (1) CWG or denim were prepared and purified; (2) the purified samples were deconstructed into a pulp; (3) to produce fibers designated by ReCell, both pulps either from cotton waste or wood pulp were combined: **ReCell-1**, pulp from fabrics washed 50 times with ECE-phosphate based detergent, to mimic the effect of domestic washing cycles; **ReCell-2**, prepared from a blend of 20% cellulose recovered after purification of treated cotton fabrics (easy care finished cotton fabric was washed 50 times with ECE-phosphate based detergent and subsequently purified in acid-alkali) and 80% wood pulp; **ReCell-Denim fibers**, pulp from waste denim was washed once with ECE-phosphate based detergent; **Lyocell**, fibers were produced from purified CWG in NMMO solution without wood pulp; Dissolution and fiber spinning: (1) pulp from different fibers was mixed with NMMO at increasing temperatures and under vacuum conditions; (2) the spinning temperature was established at 115 °C.	The surface of all studied fibers appeared to be smooth; Fibers spun from CWG had higher molecular weight than standard lyocell fibers; ReCell-2 exhibited superior mechanical and molecular properties in relation to the typical fibers regenerated from wood pulp.	[93]
Bleached softwood kraft pulp (BSWK)		Pretreatment: (1) periodate oxidation of BSWK was performed resorting to NaIO_4_ and NaCl under stirring at RT for 12 h; (2) the modified pulp was filtered and washed three times with dH_2_O; (3) modified cellulose was dispersed in NaOH solution at temperatures < 0 °C for 10 min under stirring; (4) chitosan was added to the cellulose dispersion at RT and 300 rpm for 30 min to induce the fibers crosslinking; Fiber extrusion: (1) the solution was extruded in the form of fibers into a coagulation bath of H_2_SO_4_/Na2SO_4_ at RT; (2) fibers were washed to remove excess of acid.	The fibers tenacity, in result of chitosan crosslinking, was comparable to that of viscose rayon; Crosslinked cellulose fibers become less hydrophilic, a desirable property for high-quality textile applications; Toxic CS_2_ were avoided;The entire process is water-based, simple and environmentally friendly, without requiring cellulose purification and removal of hemicellulose.	[80]
White postconsumer textiles (cotton/polyester blend)	[DBNH][OAc]	Pretreatment: (1) cotton/PES samples were shredded and blended to obtain a mixture with a concentration of 50 wt.% cotton and 50 wt.% PES; (2) the samples suffered alkaline washing to remove silicate; (3) cotton/PES blends were submitted to O_3_ and H_2_O_2_ to adjust the viscosity and to bleach the material, respectively; (4) acid washing was performed to remove the metals present; Recovery of dry-jet wet spun textile grade cellulose [M] and PES [S] fibers: (1) [M1], [S1], [S2]: cotton/PES blends were mixed with [DBNH] [OAc] for 1h at 80 °C with a concentration of cotton of 6.5 wt.%; (2) [M2]: similar conditions but higher amount of cotton, 10.5 wt.%.	Spun fibers displayed properties similar to Lyocell, with linear densities between 0.75–2.95 dtex, breaking tenacities of 27–48 cN/tex, and elongations of 7–9%; PES undergoes visible degradation once dispersed in [DBNH][OAc], this is evident by the decrease of its MMD and tensile properties.	[94]
Waste fruit peels (WFP)		Isolation of cellulose: (1) different seasonal fruits were used and fruit bran was prepared to extract cellulose; (2) to remove hemicellulose and lignin content an alkali hydrolysis was done with KOH at RT; (3) samples were bleached in NaClO_2_ at 70 °C for 1 h; (4) to disintegrate fibrils and form finest cellulose an acid hydrolysis was done with H_2_SO_4_ at 80 °C for 1 h; (5) at each step the suspension was neutralized, washed and centrifuged.	A photocatalyst cellulose/MoS_2_ was developed by *in situ* hydrothermal approach with high photocatalytic activity; Increase in photodegradation efficacy results from the existence of cellulose as support for MoS_2_, which causes a delay in the recombination of photo-generated charge carriers.	[95]
Empty fruit brunch (EFB)	LTTMs: mixture of L-malic acid-sucrose-dH_2_O at molar ratio of 2/4/2 (w/w/w) or mixture of cactus malic acid-sucrose-dH_2_O at molar ratio of 2/4/5 (w/w/w)	Delignification of EFB: (1) the EFB was pretreated with LTTMs in a ratio of 1/20 (w/w) at 80 °C for 6 h in an oil bath with magnetic stirring; (2) cellulose fibers were washed with dH_2_O for the precipitation of lignin; (3) the precipitated lignin and cellulose fibers were separated by filtration and then dried.	The EFB recovered cellulose fibers using cactus malic acid-LTTMs showed the lowest lignin content; LTTMs-delignified EFB displays a great potential for producing specialty papers for pulp and paper industries.	[96]

Abbreviations - PES: polyester; HNO_3_: nitric acid; DMSO: dimethyl sulfoxide; NaClO: sodium hypochlorite; HCl: hydrochloric acid; H_2_SO_4_: sulfuric acid; NaOH: sodium hydroxide; CH₄N₂O: urea; LiOH: lithium hydroxide; [AMIM]Cl: ionic liquid 1-allyl-3-methylimidazolium chloride; [Bmim]OAc: ionic liquid 1-butyl-3- methylimidazolium acetate; NaCl: sodium chloride; NaIO_4_: sodium (meta)periodate; CS_2_: carbon disulfide; [DBNH][OAc]: 1,5-diazabicyclo[4.3.0]non-5-enium acetate; O_3_: ozone; H_2_O_2_: hydrogen peroxide; MMD: molar mass distribution; DMCHA: N,N-dimethylcyclohexylamine; KOH: potassium hydroxide; NaClO_2:_ sodium chlorite; MoS_2_: molybdenum sulfide; LTTMs: low-transition-temperature-mixtures. Numbering in [S] samples refers to the number of washings during separation step, 1 or 2, and in [M] samples is used to distinguish between cotton contents.

**Table 2:** Production of CA from different sources.

Raw material	Solvents	Catalyst	Acetylating Agent	Methods	Observations	Ref.
Waste cotton fabrics (WCFs)		[Hmim]HSO4	(CH_3_CO)_2_O	Pretreatment: (1) WCFs were cut and shredded, and used without further purification or bleaching; Acetylation: (1) WCFs, (CH_3_CO)_2_O and 0.1–0.4 molar equivalents of ionic liquids (ILs) were mixed and heated at 100 °C for 1–5 h; (2) the mixture was poured into ethanol and stirred for 30 min; (3) the solid consisting of CA and unreacted cellulose was filtered and washed with ethanol three times and then dried at 60 °C for 24 h; (4) the sample was then refluxed for 24 h by the Soxhlet extraction method using dH_2_O; (5) the filtrate was dried in a vacuum oven at 60 °C for 24 h to obtain the water-soluble CA.	There is no water-soluble CA without an ILs catalyst; Conversion of water-soluble CA increases significantly with the increase content of ILs in a 1 h reaction time; Conversion of water-soluble CA decreases with ILs amount when the reaction time is 2, 3, 4 and 5 h. This relates to the increase of DS values and, consequent, decrease in solubility; Highest conversion was obtained with 0.2 molar equivalents of ILs in a 3 h reaction.	[107]
Cotton burr Cotton seed hull		Iodine	(CH_3_CO)_2_O	Pretreatment: (1) samples were pulverized with a hammer mill; (2) scouring step: samples were suspended in 6% solution of NaOH, heated in a water bath for 35 min, filtered, and washed with water at 95 °C; (3) bleaching step: the material was suspended in a NaOH solution at pH 12.0 with 1.5% H_2_O_2_ for 1 h, in 95 °C water bath; (4) water and caustic were removed by filtration and the pH was adjusted to 7.0; (5) the resulting powder was dried at 40 °C overnight. Acetylation: (1) samples, (CH_3_CO)_2_O and iodine were heated at 80–100 °C for 20–24 h; (2) the mixture was cooled to RT and treated with a saturated solution of Na_2_S_2_O_3_, while stirring; (3) the mixture was poured into ethanol and stirred for 30 min; (4) the solid, which contained CA, was filtered, washed and dried at 60 °C; (5) CA was dissolved in CH_2_Cl_2_ and filtered; (6) the filtrate was evaporated under vacuum at RT.	The process was optimized by varying the temperature and the amounts of (CH_3_CO)_2_O and iodine; The best yields obtained were of 15–24%, which corresponded to a conversion of 50–80% of the starting cellulose.	[116]
Rice straw (RS)		H_3_PW_12_O_40_	(CH_3_CO)_2_O	Pretreatment: (1) RS was cut and washed, dried and crushed into powder by a grinder; (2) powder was Soxhlet extracted using a toluene-ethanol mixture for 24 h to remove wax, pigments and oils, followed by drying; (3) the dewaxed powder was stirred in KOH solution with H_2_O_2_; (4) the mixture was then cooled to RT, filtered and washed until the filtrate became neutral, and finally dried. Acetylation: (1) samples, CH_3_COOH, (CH_3_CO)_2_O, CH_2_Cl_2_, and H_3_PW_12_O_40_ were mixed; (2) the mixture was refluxed; (3) the mixture was filtered and the residue collected; (4) acetone was added, the material was filtered and the filtrate was evaporated after stirring; (5) the solid was dried overnight at 80 °C.	83 wt.% content of cellulose was obtained after pretreatment with 4% KOH and immersion in CH_3_COOH for 5 h; Acetone-soluble CA with DS values around 2.2 were obtained by changing the amount of H_3_PW_12_O_40_ and the acetylation time.	[117]
Green landscaping waste (GLW)	CH_3_COOH	H_2_SO_4_	(CH_3_CO)_2_O	Pretreatment: (1) GLWs and H_3_PO_4_ solution were loaded into a reactor at 150 °C for 15 min and under stirring to carry out the hydrolysis process; (2) the final product was filtered. Acetylation: (1) CH_3_COOH, (CH_3_CO)_2_O and H_2_SO_4_ were mixed with GLWs; (2) the mixture was heated to 60 °C under stirring; (3) the reacted mixture was cooled to RT, filtered and evaporated to recover CA; (4) CA was dried at 80 °C for 12 h.	Diluted H_3_PO_4_ disrupted the crystalline structure of cellulose and increased the amorphous region, rendering the cellulose more accessible to (CH_3_CO)_2_O, leading to a more effective acylation; Acetylation of pinewood without pretreatment registered an 8.3% yield of CA (low); High acetylation levels were obtained with pretreatment at 150 °C, 1.8 h, 8 mL/g, 100 mL, 1.67 wt.% of H_3_PO_4_ in solution, and 150 rpm.	[113]
Microcrystalline cellulose (MCC) Cotton linter pulp Wheat straw pulp Bamboo pulp Bleached softwood sulfite dissolving pulp Bleached hardwood kraft pulp (HP)	DMSO	NaOH	C_4_H_6_O_2_	Pretreatment: delignification with NaClO_2_ and KOH; Acetylation (transesterification): (1) cellulose was dissolved in DMSO; (2) NaOH was added dropwise to activate the -OH groups; (3) C_4_H_6_O_2_ was poured into the mixture under stirring for 15 min to obtain CA.	Cellulose was esterified within 15 min; CA-MCC solution displayed the lowest viscosity, while the CA-HP solution had the highest values, showing also higher DPs, which hindered the DS; DS values for all CA samples were above 2.52, confirming a successful synthesis; - Most of the obtained fibers were triacetate fibers with DS higher than 2.75; CA fibers with high DPs exhibited the lowest DS; The yields of the obtained subtracts were: CA-MCC 89.21%, CA-CP 84.75%, CA-WP 72.38%, CA-BP 68.83%, CA-SP 66.28%, and CA-HP 58.59%.	[21]
Babassu coconut shells (BCS)	CH_3_COOH	H_2_SO_4_	(CH_3_CO)_2_O	Pretreatment (organosolv process): (1) pretreatment of endocarp of BCS; (2) reaction of raw material with 80% ethanol/20% HNO_3_ v/v for 3 h under reflux (at ≈ 100 °C); (3) reaction with NaOH for 1 h at RT; (4) obtained samples were washed to reach pH 7.0. Acetylation: (1) CH_3_COOH was added to the obtained cellulose (30 m at RT); (2) H_2_SO_4_ was added and stirred for 25 min, followed by the addition of (CH_3_CO)_2_O which was stirred for the same time; (3) stirring for 24 h at RT; (4) water was added to stop the reaction, the precipitated CA was filtered and washed with dH_2_O; (5) neutralization with 10% Na_2_CO_3_ (pH 7.0); (6) CA was washed for 2 days using dialysis tubing (water replaced every 6 h) and dried at 90 °C for 4 h.	The organosolv extraction was rapid, effective (with yields of 70–95%) and eco-friendly;The yield of the acetylation reaction was estimated in 76%; The CA DS was determined at 2.63 ± 0.01.	[110]
Sugarcane straw (SCS)	Glacial CH_3_COOH	H_2_SO_4_	(CH_3_CO)_2_O	Pretreatment: (1) (acid) SCS was treated with H_2_SO_4_ (10% v/v) at 100 °C for 1 h; (2) (alkaline) SCS was treated with NaOH (5% w/v) at 100°C for 1 h; (3) (chelating) SCS was treated with 0.5% C_10_H_16_N_2_O_8_ for 30 min at 70 °C; (4) (bleaching) SCS was treated with 5% (v/v) H_2_O_2_ and 0.1% MgSO_4_. Acetylation: (1) CH_3_COOH was added to SCS cellulose and stirred at 37.8 °C for 1 h; (2) glacial CH_3_COOH and H_2_SO_4_ were added to the mixture for 45 min; (3) (CH_3_CO)_2_O and H_2_SO_4_ were added after the mixture was cooled to 18.3 °C; (4) the temperature was increased to 35 °C and the mixture was stirred for 1.5 h; (5) water and glacial CH_3_COOH were added and stirred for 1 h; (6) the material obtained was washed with dH_2_O until reaching pH 7.0.	Cellulose with 90% purity was obtained; CA presented a DS of 2.72 ± 0.19 and a percentage of acetyl groups of 41.05 ± 2.77%, characteristic of a triacetate.	[106]
Sorghum straw (SS)	CH_3_COOH	H_2_SO_4_	(CH_3_CO)_2_O	Pretreatment (extraction): different cooking times (1.5–2.5 h) and alkali solutions (NaOH) (0.75–1.25% w/v) were applied at a ratio of 1/20 (w/v) of SS/NaOH at 90 °C; (2) samples were washed several times with dH_2_O until NaOH was completely removed, followed by drying at 50 °C for 12 h in oven; (3) (bleaching) SS acetate buffer (pH 4.5) and 2 wt.% NaClO_2_ were combined at 80 °C for 0–35 min and 20–25 mL; (4) samples were dried at 50 °C for 12 h. Acetylation: time ranged from 6 to 16 h; (1) bleached pulp was added to CH_3_COOH solution; (2) after 30 min, H_2_SO_4_ and (CH_3_CO)_2_O were added and stirred for 25 min; (3) (CH_3_CO)_2_O was added to the mixture and stirred for 30 min; (4) the mixture was left to rest for 6, 7, 8, 9, 10, 11, 13, 15 and 16 h, at 25 °C; (5) CA was precipitated in water and filtered; (6) the material was washed to remove the excess of CH_3_COOH.	CA with the highest DS was obtained by acetylating cellulose with (CH_3_CO)_2_O for 16 h at RT; CA reached a DS of 2.6–2.7.	[109]
Microfibrillated date seeds cellulose	CH_3_COOH	H_2_SO_4_	(CH_3_CO)_2_O	Acetylation: (1) (swelling) seeds were mixed with CH_3_COOH at RT for 2 h; (2) the mixture was poured in a cooled solution of (CH_3_CO)_2_O, CH_3_COOH and H_2_SO_4_; (3) dH_2_O was poured to the reaction at constant stirring to precipitate CA; (4) the residue was washed with dH_2_O until neutral pH was reached; (5) the obtained material was dried in an air oven at 50 °C.	A yield of 79% was obtained for cellulose triacetate.	[118]
Untreated sisal Treated sisal (mercerized) Mercerized cotton linters	DMAc/LiCl		(CH_3_CO)_2_O	Pretreatment (mercerization): (1) samples were mercerized in 20% NaOH solution at 0 °C for 1 h; (2) alkali-swollen material was washed in dH_2_O until a constant pH was reached. Acetylation: (1) cellulose and DMAc were mixed, heated at 150 °C and stirred for 1 h; (2) LiCl was added and the mixture was heated to 170 °C; (3) (CH_3_CO)_2_O was added dropwise at 110 °C for 1 or 4 h; (4) precipitation was induced with CH_3_OH followed by purification via Soxhlet extraction and drying at 50 °C.	LiCl did not influence the DS but affected aggregation during filtration; High LiCl content induced separation of the cellulose chains, which in turn reduced aggregation; Mercerized products reached higher DS values than untreated samples.	[114]
Waste polyester/cotton blended fabrics (WBFs)		[Hmim]HSO_4_	(CH_3_CO)_2_O	Pretreatment: (1) WBFs were cut and shredded. Acetylation: (1) (CH_3_CO)_2_O and [Hmim]HSO_4_ were added to WBFs powders at 100 °C for 12 h; (2) the mixture was poured into ethanol; (3) the solid, which consisted of CA and PET was filtered, washed and dried; (4) to extract acetone-soluble CA, part of the sample was refluxed using acetone; (5) the filtrate was dried and refluxed using DMF.	[Hmim]HSO_4_ at 0.4 molar equivalents of IL was the most acetone-soluble formulation; The extraction yield of acetone-soluble CA was 49.3%, which corresponded to a conversion of 84.5% of WBFs original cellulose; 96.2% of the original PET were recovered.	[111]
Sugarcane bagasse (SB)	CH_3_COOH	H_2_SO_4_	(CH_3_CO)_2_O	Pretreatment (purification): (1) the material was mixed with NaOH at RT for 18 h; (2) the mixture was filtered and washed with dH_2_O; (3) the material was refluxed in a HNO_3_/ethanol solution at 20% v/v for 3 h (solution changed every hour); (4) the bagasse was washed with dH_2_O and oven dried at 105 °C for 3 h; Acetylation: (1) SB was mixed with CH_3_COOH and stirred for 30 min; (2) H_2_SO_4_ and CH_3_COOH were added to the system; (3) the mixture was filtered and (CH_3_CO)_2_O was added; (4) the solution was returned to the bagasse container and stirred for 30 min; (5) the mixture stood at 28 °C and dH_2_O was added to stop the reaction and precipitate CA; (6) CA was washed in dH_2_O and dried at RT overnight.	After sugarcane bagasse purification, 75% of α-cellulose was attained; The CA viscosity-average molecular weight increased from 5.5 × 10^3^ to 55.5 × 10^3^ g/mol.	[108]
Commercial cellulose	DMSO/TBAF	CDI	C_11_H_16_O_2_, CH_3_COOH, C_18_H_36_O_2_, and C_5_H_4_O_3_	Acetylation: (1) esterification of cellulose using carboxylic acids, activated in situ with CDI; (2) 15 min at RT was enough to obtain a clear solution.	Cellulose esters were prepared with DS values up to 1.9, without any required pretreatment; Esterification with C_11_H_16_O_2_, and C_5_H_4_O_3_ was the most effective.	[119]

Abbreviations - DP: degree of polymerization; DS: degree of substitution; Ic: crystallinity index; [Hmim]HSO_4_: N-methyl-imidazolium bisulfate; (CH_3_CO)_2_O: acetic anhydride; NaOH: sodium hydroxide; H_2_O_2_: hydrogen peroxide; Na_2_S_2_O_3_: sodium thiosulfate; CH_2_Cl_2_: dichloromethane; KOH: potassium hydroxide; CH_3_COOH: acetic acid; H_3_PW_12_O_40_: phosphotungstic acid; H_2_SO_4_: sulfuric acid; NaClO_2_: sodium chlorite; H_3_PO_4_: phosphoric acid; C_4_H_6_O_2_: vinyl acetate; HNO_3_: nitric acid; Na_2_CO_3_: *s*odium carbonate; C_10_H_16_N_2_O_8_: ethylenediamine tetraacetic acid; MgSO_4_: magnesium sulfate; LiCl: lithium chloride; DMAc: N,N-dimethylacetamide; DMF: dimethylformamide; DMSO: dimethyl sulfoxide; TBAF: tetrabutylammonium fluoride trihydrate; CDI: carbonyldiimidazole; C_11_H_16_O_2_: adamantane carboxylic acid; C_18_H_36_O_2_: stearic acid; C_5_H_4_O_3_: 2-furancarboxylic acid.

**Table 3:** Production of nanocelluloses (CNF, CNCs, and BC) from different sources.

Type	Raw material	Main Agent	Methods	Observations	Ref.
CNF	Wheat straw (WS) Waste wheat straw (WWS)	*p*-TsOH	Fractionation of WS and WWS using *p*-TsOH: (1) WS or WWS were added to the concentrated acid solution at continuous stirring; (2) after, it was filtered. Mechanical fibrillation: (1) two hydrolyzed fiber samples were mechanically fibrillated to produce LCNF. Alkaline peroxide post-treatment: (1) bleaching was conducted at 60 °C by adding the obtained LCNF suspension to a H_2_O_2_ solution (stirring); (2) the pH of the suspension was adjusted to 11.5 with 4 M NaOH; (3) the resultant purified LCNF (P-LCNF) was dialyzed using dH_2_O until the pH was constant.	Alkaline peroxide post-treatment was further conducted to obtain purified lignocellulosic nanofibrils (P-LCNF) with low lignin content and thin diameters; The low-temperature fractionation process on WS and WWS fibers could yield cellulose nanomaterials with potential value-added for a variety of applications and uncover a new efficient processing tool for agricultural wastes.	[135]
	Arecanut husk (AH)	HCl, NaOH	Isolation of cellulose nanofibrils: (1) the dried AH fibers were dewaxed with a mixture of toluene and ethanol for 48 h at 50 °C, followed by washing with boiling water and dried in air; (2) the dried fibers were then cut; (3) to remove lignin and hemicelluloses, a treatment with NaOH was applied at 50 °C for 4 h; (4) samples were washed to remove the alkali compounds and treated with HCl to break the cell walls and separate the microfibrils; (5) fibers were washed with dH_2_O to eliminate any acid traces; (6) fibers were grinded into a pulp form and treated again with alkali to remove the remaining non-cellulosic components, followed by acid hydrolysis; (7) the delignification was further carried out by bleaching with NaClO_2_ and glacial acetic acid for 2 h at 60 °C.	Highly crystalline and thermally stable cellulose nanofibrils, with very high aspect ratio, were prepared from AH fibers by HCl hydrolysis followed by mechanical fibrillation.	[136]
	Softwood sulfite pulp (SSP) Wheat straw (WSP1) Refined fibrous wheat straw cellulose suspension (WSP2) Refined beech wood (BWP1) Refined fibrous beech wood pulp suspension (BWP2)		Mechanical pretreatment: (1) SSP, WSP1 and WSP2 were milled; Mechanical high-shear disintegration: (1) mechanical treatment under high pressure was performed to separate the nanofibrillated cellulose from the suspensions.	The homogeneity of the NFC material was determined as more important for its reinforcement potential than the DP.	[137]
	Waste jute bags (WJB)	Toluene/ethanol, NaOH, C_2_H_6_O, H_2_O_2_, HCl	Pretreatment (isolation of lignin and cellulose nanofibrils): (1) the WJB were chopped into small pieces, washed and dried; (2) the samples were dewaxed in a soxhlet apparatus using toluene/ethanol; Lignin and cellulose removal: (1) the pretreated jute fibers were subjected to soda cooking at high temperatures; (2) temperature was reduced to separate the fibers; (3) to precipitate lignin the pH was lowered and the samples filtered; (4) the mixture was subjected to C_2_H_6_O solution to increase its purity by dissolving the hemicellulose; (5) jute fibers pulp were bleached with H_2_O_2_ and the residual lignin dissolved; (6) bleached pulp was hydrolyzed with HCl resulting in defibrillation of the cellulose.	It was possible to isolate cellulose nanofibrils and extract lignin by discarding the hemicellulose using a soda cooking pretreatment followed by fiber defibrillation by acidic hydrolysis.	[138]
CNCs	Waste polyester/cotton blended fabrics (WBFs)	H_3_PW_12_O_40_	Separation treatment: (1) the WBFs were mixed with H_3_PW_12_O_40_ aqueous solution and heated to 120–170 °C for 3–8 h; (2) the solution was filtered and MCC were oven-dried in a vacuum oven at 60 °C for 6 h, and stored for further processing.	The optimal conditions for the separation treatment were determined as follows: 3.47 mmol/L of HPW concentration, solid/liquid ratio of 1/20, reaction temperature of 140 °C, and reaction time of 6 h; The yields of MCC and PES were 85.12% and 99.77%, respectively.	[139]
	Pineapple leaf (PL)	H_2_SO_4_	Pretreatment: (1) raw PL was ground; (2) the powder was treated with a NaOH aqueous solution for 4 h at 100 °C; (3) samples were bleached in acetate buffer and NaClO_2_ at 80 °C for 4 h; Isolation of cellulose nanocrystals: (1) treated PL was milled with a blender; (2) the samples were submitted to hydrolysis at 45 °C for 5 min in H_2_SO_4_; (3) the resulting suspension was ultrasonicated for 10 min and stored at 4 °C.	The most successful extraction of high crystalline cellulose was attained with a hydrolysis process of 30 min.	[140]
	Seaweed	H_2_SO_4_C_6_H_11_ClN_2_	Pretreatment: (1) the powdered seaweed samples were treated with NaOH under microwave irradiation for 30 min at 360 W; (2) to ensure complete delignification, the alkali-pretreated sample was bleached using H_2_O_2_ for 4 h at 55 °C; (3) the bleached sample was subjected to hydrolysis using H_2_SO_4_ and C_6_H_11_ClN_2_ for 30 min at 95 °C to remove the amorphous parts of the sample.	CNCs can be successfully isolated from *Gelidiella aceroso* via microwave irradiation, which is an alternative energy source for alkali treatment.	[141]
	Groundnut shells (GNS)	H_2_SO_4_	Pretreatment: (1) GNS were cleaned by washing in dH_2_O, dried and milled; (2) powdered shells were submitted to soxhlet extraction for 8 h using benzene/methanol; (3) the dewaxed shells were bleached with NaClO_2_ to remove lignin at 70 °C for 2 h, and then filtered; (4) the holocellulose obtained was treated with 1 M NaOH solution at 65 °C for 2 h to remove hemicelluloses; (5) the extracted product was dried for 24 h at 100 °C; Isolation of cellulose nanocrystals: (1) a certain amount of cellulose was treated with H_2_SO_4_ for 75 min at 45 °C; (2) in the end the samples were washed.	CNCs were successfully isolated from groundnut shells, after purification and acid hydrolysis treatment, reaching a yield of 12%.	[142]
BC	Undyed cotton-based textile wastes	[AMIM]Cl	Pretreatment: (1) the waste cotton was cut into small pieces; (2) these were added to an IL solution at 90, 110 or 130 °C; (3) dH_2_O was used as an anti-solvent for regenerated cellulose; Enzymatic hydrolysis: (1) cellulose regenerated and untreated cotton were immersed in citrate buffer containing cellulase and incubated at 50 °C; (2) the amount of IL affecting the polymer yield was analyzed.	Pretreatment with [AMIM]Cl is very efficient in increasing the hydrolytic rate of cotton cloth, since after 4 h the yields of the reduced sugar from pretreated and untreated cotton cloth were 22.4% and 4.0%, respectively; Higher BC yields (40–65%) were obtained in cotton enzymatic hydrolysate cultures; BC production decreased at IL concentration of 0.001 g/mL.	[143]
	Potato peel waste (PPW)	HNO_3_; H_2_SO_4_; HCl; H_3_PO_4_	Production of PPW acid hydrolysate: (1) PPW was added to solutions of HNO_3_, H_2_SO_4_, HCl and H_3_PO_4_ at 100 °C for 2, 3, 4 and 6 h; (2) the pH of each mixture was neutralized to 6 with 1 M NaOH; PPW as alternative media for BC production: five factors were tested to optimize BC production, initial pH (7–11), media volume (mL), inoculum size (4–12%), temperature (25–45 °C), and incubation time (2–6 days); BC purification: (1) the produced BC was collected, rinsed in dH_2_O, and immersed in 1 N NaOH at 60 °C for 90 min to remove attached cells and impurities; (2) pellicles were rinsed with methanol, washed with the dH_2_O and dried at 60 °C for 24 h.	Maximum BC yield was achieved using PPW-nitric acid hydrolysate at 2.61 g/L followed by PPW-sulfuric acid hydrolysate at 2.18 g/L; Optimal BC production conditions were determined as pH 9 with 8% inoculum size and volume of 55 mL, at 35 °C and incubation of 6 days.	[144]
	Wheat straw (WS)	[AMIM]Cl	Pretreatment: (1) WS was mixed with IL; (2) the mixture was heated from 90 to 120 °C and incubated for different times under 500 rpm; (3) dH_2_O was added to straw/IL solution to regenerate the straw; Enzymatic hydrolysis: (1) WS regenerated was placed in acetate buffer (pH 5.0) containing cellulase and was incubated at 50 °C at 80 rpm.	The hydrolytic efficiency of regenerated straw increased compared to untreated materials; The yield of the straw was 71.2% after pretreatment in [AMIM]Cl at 110 °C for 1.5 h, with a 3 wt.% straw dosage, which was 3.6 times higher than that of untreated straw (19.6%); BC yield obtained from straw hydrolysates was higher than that from glucose-based media.	[145]
	Kitchen waste (KW)	α-amylase; amylglucosidase	Pretreatment: (1) samples were subjected to a washing process using tap water to separate the KW into solid fraction (starch-rich solid) and liquid fraction (oil/water mixture); (2) the solid fraction was sterilized at 121 °C for 15 min; Enzymatic saccharification of the solid fraction: (1) samples were hydrolyzed using α-amylase and amylglucosidase at 55 °C for 24 h, at 150 rpm; BC production: (1) the glucose concentration of the resultant hydrolysate was diluted to 50 g/L; (2) then 5 g/L peptone, 5 g/L yeast extract, 1.15 g/L citric acid and 2.7 g/L disodium hydrogen phosphate were added to prepare the BC production media; (3) the seed culture was incubated at 30 °C and 150 rpm for 2 days; (4) 10 mL of the cultured seed were inoculated in 100 mL of production media (pH of 5.0), which was cultivated at 30 °C under static conditions for 15 days; (5) at 1, 4, 8, 12 and 15 days the concentrations of glucose and glycerol were measured.	The washing with dH_2_O during pretreatment removed oil and NaCl from samples, increasing the BC yield.	[146]

Abbreviations - DP: degree of polymerization; H_3_PW_12_O_40_: phosphotungstic acid; NaOH: sodium hydroxide; NaClO_2:_ sodium chlorite; H_2_SO_4:_ sulfuric acid; C_6_H_11_ClN_2_: 1-ethyl 3-methyleimiddazoliumchloride; *p*-TsOH: *p*-toluenesulfonic acid; HCl: hydrochloric acid; C_2_H_6_O: ethanol; HNO_3_: nitric acid; H_3_PO_4_: phosphoric acid; [AMIM]Cl: 1-allyl-3-methylimidazolium chloride; LCNF: lignocellulosic nanofibrils.

**Table 4:** Examples of compounds incorporated in electrospun nanostructures containing cellulose or its derivatives.

Subtract	Drugs	Nanoparticles	Natural Extracts	Ref.
Cellulose	Tetracycline hydrochloride (TH) Ciprofloxacin (CIF) Donepezil hydrochloride (DNP)	Silver NPs (AgNPs) Zinc oxide NPs (ZnONPs)	Bromelain	[159,160,161,162,163,164,165]
CA	TH Ferulic acid (FA) Ibuprofen (IBU) Ketoprofen (KET) Amoxicillin Thymoquinone (TQ) Silver salt of sulfadiazine (SSD)	Silver Titanium dioxide Zinc oxide Copper	Cinnamon (CN); Lemongrass (LG); Peppermint (PM) Rosemary; Oregano Thymol Zein Asiaticoside (AC) Curcumin (Curc) Acid gallic Gingerol Garlic extract	[60,65,67]; [166,167,168,169,170,171,172,173,174,175,176,177,178,179,180,181,182]
CNC	TH	ZnO AgNPs	Thymol	[183,184,185,186,187]; [188,189]
BC		Soy protein particles Graphene oxide (GO)	Tragacanth gum (TG)	[190,191,192]

**Table 5:** Processing of cellulose-, CA- and nanocellulose-containing electrospun mats incorporated with drug molecules.

Drugs	Polymer(s) and solvent(s)	Processing conditions	Observations	Ref.
**Cellulose**				
TH	3% w/v of TCMC in DMF; 1% w/v of PEO in CHCl₃	Single nozzle and core-shell electrospinning; Graft copolymerization: NaCMC was grafted with MA originating NaCMC-co-MA copolymer (TCMC); Single nozzle: 5% w/w TH (in relation to methanol concentration) was added to TCMC/PEO and processed at 15 kV, distance of 20 cm and feed rate of 3 mL/h; Core-shell: TCMC was used at the shell and 5% w/w TH/PEO was used at the core, fibers were produced using potential of 15 kV, distance of 18 cm and feed rate of 0.4 mL/h.	Fibers produced from polymer blend were more uniform and bead free than those generated from core-shell; The TH release profile in core-shell nanofibers was more efficient, with an initial burst release of only 26% (first 30 min), and a 92% released within 72 h; TH-loaded TCMC/PEO core-shell nanofibers revealed excellent antibacterial effects against Gram-positive bacteria.	[159]
CIF	13% w/v of EC or PVP in HFIP	Single nozzle electrospinning; 5% and 15% w/w of CIF (with respect to the polymer concentration) was added to PVP and to EC; Fibers were produced using potential of 20 kV, distance of 16 cm and a feed rate of 0.8 mL/h. Fibers were collected from an aluminum foil and from a gauze covering the foil. The following samples were produced: S1: control with PVP; S2: PVP/CIF (5%) in foil; S2G: PVP/CIF (5%) in gauze; S3: PVP/CIF (15%) in foil; S3G: PVP/CIF (15%) in gauze; S4: control of EC; S5: EC/CIF (5%) in foil; S5G: EC/CIF (5%) in gauze; S6: EC/CIF (15%) in foil; S6G: EC/CIF (15%) in gauze.	Neat PVP fibers generated the largest diameters (832 ± 241 nm), which decreased after CIF addition; Neat EC fibers displayed diameters of 597 ± 214 nm; while S5 and S6 attained diameters of 435 ± 137 nm and 368 ± 108 nm, respectively; Drug release was slower on EC than on PVP fibers; After 480 min, both sets of fibers had released 90% of their CIF loading; Samples showed no toxicity towards cells; Inhibition zones of the CIF-loaded PVP fibers (S2 and S3) for *E. coli* and *S. aureus* after 24 h contact were 5.30–5.71 cm and for CIF-loaded EC fibers were 4.29–4.72 cm.	[160]
DNP	12.5% w/v of PU in DMF; 1.2, 2.5, 5.0, and 10.0% w/v of HPC in DMF	Single nozzle electrospinning; PU was blended with various concentrations of HPC and DNP at 1.25% w/v (RT); Fibers were produced using potential of 15 kV, distance of 15 cm and a feed rates of 1.0 mL/h.	Mats presented a uniform, non-beaded, and smooth morphology, with diameters ranging from 464 ± 24 to 995 ± 14 nm; PU/HPC/DNP mats portrayed generally smooth nanofibers, with the exception of ratios 10/4/1 and 10/8/1 which displayed some beads; Nanofibers composed of PU/HPC/DNP at ratios 10/0/1, 10/1/1, 10/2/1, and 10/4/1 revealed an initial burst release of 66, 66, 61, and 71%, respectively; The total amount of DNP on the fibers ranged 85–90%;In vitro cytotoxicity analysis indicated that PU/HPC mats were well tolerated by the skin and the DNP was not irritant.	[161]
**Cellulose acetate**				
TH	18% w/w CA in acetone/ DMAc at 2/1 v/v; 10% w/w PCL in DMF/ THF at 1/1 v/v; CA/PCL were mixed at 1/1, 2/1 and 3/1 v/v; 1% w/w dextran was added to CA/PCL	Single nozzle electrospinning; 1% w/w THC was added to CA/PCL/dextran; Fibers were produced using potential of 15 kV, distance of 15 cm and feed rate of 1.0 mL/h.	Fiber diameters varied from 0.28 to 2.20 µm; The CA/PCL/Dextran/THF were very smooth; Higher amounts of PCL produced more uniform fibers; Fibers modified with dextran were dense, uniform and revealed smaller diameters; THC loaded nanofibers were very biocompatibility, accelerating 3T3 fibroblasts proliferation and differentiation; Drug loaded mats were effective against *S. aureus* and *E. coli* bacteria.	[60]
FA	Core: 16% w/v of gliadin in HFIP/TFA at 8/2 v/v; Middle layer: 6% w/v CA in acetone/acetic acid at 2/1 v/v; Outer layer: acetone and acetate acid at 2/1 v/v	Triaxial electrospinning; FA: 4% w/v in 8/2 v/v HFIP/TFA and mixture with the 16% w/v gliadin (core); Four different fibers were produced using potential of 15 kV, distance of 20 cm and feed rates of 0.3 outer, 0.1–0.5 middle and 2 inner.	Fibers were linear, cylindrical and with a smooth surface; As feed rates increased diameters decreased and the sheath thickness decreased; Thicker CA coatings increased the release time; The sheath prevented the initial burst release; After the first hour, continued drug release was still observed.	[166]
IBU	Core: 16% w/v of gliadin in HFIP/TFA at 8/2 v/v; Middle layer: 0, 1, 3 and 5% w/v CA in acetone/acetic acid at 2/1 v/v; Outer layer: acetone and acetate acid at 2/1 v/v	Triaxial electrospinning; IBU: 4% w/v in 8/2 v/v HFIP/TFA and mixture with the 16% w/v gliadin (core); Four different fibers were produced using potential of 15 kV, distance of 20 cm and feed rates of 0.3 outer, 0.3 middle and 2 inner.	Fibers were linear, cylindrical and with a smooth surface; Diameters increased with the increased content of CA in the middle layer: 540 (0%), 660 (1%), 720 (3%), and 870 (5%) nm; Higher CA concentrations also increased the sheath thickness to 1.82 (1%), 5.85 (3%), and 11.60 (5%) nm; Time for IBU complete release increases with the fiber sheath thickness; In the first hour, release of IBU was determined at 34.2 ± 4.5% (0%), 8.3 ± 4.6% (1%), 5.4 ± 4.1% (3%), and 2.7 ± 3.1% (5%).	[169]
KET	Core and Sheath: 11% w/v CA in acetone/DMAc/ethanol at 4/1/1 v/v	Coaxial electrospinning; KET: 2% w/v (in relation to the polymers mass) was mixture with 11% w/v CA; Fibers were produced using potential of 15 kV, distance of 15 cm and a feed rate at the core of 1.0 mL/h and at the sheath at 0.0, 0.2 and 0.4 mL/h.	As the feed rate at the sheath increased the diameters decreased and the fibers became smoother and uniform; Fiber produced with a 0.2 mL/h feeding rate averaged 240 nm and were capable of sustaining a more controlled release profile of KET.	[171]
Amoxicillin	8% w/v CA in acetone/water at 80/20 v/v 8% w/v PVP in ethanol/water at 85/15 v/v.	Coaxial electrospinning; Two different nanofibers were produced: CA/PVP/CA: PVP-core and PVP/CA/PVP: CA-core; Fibers were produced using potential of 15 kV, distance of 15 cm and a feed rates between 0.3 and 1.0 mL/h; After electrospinning, dried rectangular-shaped samples were immersed in a 1 M aqueous solution of amoxicillin for 90 min.	CA/PVP/CA after being washed in water showed the existence of cylindrical fibers; PVP/CA/PVP washed with water showed lower diameters (due to dissolution of PVP); Fibers diameters ranged from 0.5 to 2.0 μm; Young’s Modulus and the strain at break of CA/PVP/CA are slightly higher than PVP/CA/PVA; Drug release kinetics was dependent on the media pH; Time release of amoxicillin was of ≈ 15 days and was accelerated at basic pHs (pH = 7.2).	[173]
TQ	6% w/v PLA/CA in DCM/DMF at 7/3 v/v, at ratios 9/1 and 7/3 w/w	Single nozzle electrospinning; 3% w/w TQ (in relation to the polymers mass) was mixture with PLA/CA; Fibers were produced using potentials of 20–24 kV and feed rates of 1.5–3.0 mL/h.	Fiber diameters reduced with increased CA content; Presence of TQ reduced even more the diameters; 7/3 PLA/CA loaded with TQ revealed the most porous structure, with an initial burst of TQ that lasted 24 h, followed by a more sustained release of the drug for 9 successive days; 7/3 PLA/CA loaded with TQ promoted the most fibroblasts proliferation and collagen deposition and was the most effective against bacteria.	[175]
SSD	24% w/w CA in DMF/acetone at 6/4 v/v	Single nozzle electrospinning; SSD was mixed with CA solution at 0.125, 0.25, 0.37 and 0.50% w/w; Fibers were produced using potential of 12 kV and distance of 15 cm.	SSD was uniformly distributed along the fibers; The average fiber diameters decreased with the increasing loading of SSD, from ≈ 292 nm to ≈ 286 nm; 0.5% w/w SSD was the most effective concentration against bacteria.	[176]
**Cellulose Nanocrystalline**				
TH	10% w/v PHBV in chloroform/DMF at 9/1 w/w	Single nozzle electrospinning; 1, 3, 6, 9 and 10% w/w CNCs were added to the PHBV solution; 5, 15, and 25% w/w TH were added to the PHBV/CNCs solutions; Fibers were produced using potential of 15 kV with a distance of 18 cm and a feed rate of 1.0 mL/h (during 6 h).	Addition of 3 to 6% w/w CNCs to the PHBV nanofibers (1025 ± 96 nm) decreased the fibers from 748 ± 62 to 620 ± 33 nm, respectively; The tensile strength and Young’s modulus increased with the increased CNCs content, and reached a maximum with 6% w/w CNCs; The higher CNCs content improved the hydrophilicity of PHBV nanocomposite; The percentage of drug loaded and the loading efficiency were 25.0 and 98.8%, respectively (≈ 86% HF was delivered within 540 h for nanofibrous containing 6% w/w CNCs).	[183]
	16% w/w PCL in acetic acid/dH2O 90/10 v/v	Single nozzle electrospinning; Synthesis of CNC: (1) high molecular weight cellulose was extracted from cotton waste; (2) cellulose was hydrolyzed in H2SO4; 1% w/w TH was dissolved in 90% acetic acid; 0, 0.5, 1.0, 1.5, 2.5, 4% CNCs were added to the TH solution and then mixed with PCL; Fibers were produced using potential of 17 kV with a distance of 16 cm and a feed rate of 0.9 mL/h.	The lowest fiber diameters were obtained with 4% CNCs; The highest tensile stress was obtained was with 1.5% CNCs; During biodegradation studies the weight loss of CNCs-incorporated samples was much higher than for pure PCL nanofibers; Drug release was slower with increasing amounts of CNCs in the PCL nanofibers.	[184]
	10% w/w PLA in chloroform/DMF at 9/1 w/w	Single nozzle electrospinning; Synthesis of CNC: MCC was hydrolyzed in H2SO4; PEG/CNCs were mixed at 1/1; PLA was mixed with PEG/CNCs at 1–10% w/w; 3, 10, 15, 20 and 30% w/w TH were added to the polymeric blend; Fibers were produced using potential of 18 kV with a distance of 15 cm and a feed rate of 1 mL/h.	The diameter of the PLA nanofibers was 2.5 ± 0.1 µm and decreased to 1.2 ± 0.1 µm with the addition of 10% w/w PEG/CNCs; Increased drug loading reduced the fibers diameters; The water contact angle was significantly reduced with the incorporation of 10% w/w PEG/CNCs; Composite nanofibers containing 15–30% TH delivered more than 95.7% of their content within 1032 h, while neat PLA nanofibers only released 13% of the drug; Composite nanofibers showed good biocompatibility with MG63 cells.	[185]

Abbreviations - EC: ethyl cellulose; PVP: polyvinylpyrrolidone; CHCl_3:_ chloroform; HFIP: 1,1,1,3,3,3-hexafluoro-2-propanol; TCMC: thermoplastic carboxymethyl cellulose; PEO: poly(ethylene oxide); HPC: hydroxypropyl cellulose; PU: polyurethane; DNP: donepezil hydrochloride; TFA: trifluoroacetic acid; DMAc: dimethylacetamide; DMF: dimethylformamide; THF: tetrahydrofuran; PCL: polycaprolactone; PLA: polylactic acid; DCM: dichloromethane; PHBV: poly(3- hydroxybutyrate-co-3-hydroxyvalerate); MCC: microcrystalline cellulose; PEG: polyethylene glycol.

**Table 6:** Processing of cellulose-, CA- and nanocellulose-containing electrospun mats incorporated with nanoparticles.

Nanoparticles	Polymer(s) and solvent(s)	Processing conditions	Results	Ref.
**Cellulose**				
AgNPs	4% w/v CMC and 4% PEO w/v in water	Single nozzle electrospinning; Fibers were produced using potential of 22 kV, with distance of 15 cm and feed rate of 2 mL/h; After, electrospinning CMC/PEO mats were carefully immersed in AgNO_3_ solution (0.1 mol/L, to substitute Na+ with Ag+) and irradiated with UV-light.	The average diameter of CMC/AgNPs fibers (89 ± 23 nm) was smaller than that of CMC/PEO fibers (103 ± 30 nm); CMC/AgNPs nanofiber mats were 100% effective against *S. aureus* and *E. coli.*	[162]
	17% w/w CA in DMF/acetone at 1/2 v/v	Single nozzle electrospinning; Cellulose nanofibers were prepared from CA nanofibrous mats by a simple alkaline treatment with NaOH and coated with silver by immersion in AgNO_3_, forming CEAgNP; Fibers were produced using potential of 15 kV, with distance of 15 cm and feed rate of 0.06 mL/h.	CA nanofibers showed a smooth and regular morphology with an average diameter of 291 nm, and cellulose displayed diameters averaging 289 nm; All CEAgNP samples were 100% bactericidal, being effective in preventing growth of *E. coli* and *S. aureus* strains.	[163]
ZnO NPs	2% w/v CMC and 10% w/v PVA/dH_2_O	Single nozzle electrospinning; 1/1 w/w PVA/CMC was combined with 3% w/w of ZnO NPs (relative to PVA/CMC blend) and then with EM at 5% w/w (relative to PVA/CMC blend) and mixed until a homogenous mixture was obtained; Fibers were produced using potential of 16 kV, with distance of 20 cm and feed rate of 0.3 mL/h; - Crosslinking was performed with 2% glutaraldehyde vapor in a desiccator for 48 h and then dipped in 3% AlCl_3_ in ethanol.	PVA/CMC nanofibers ranged 214.5 ± 26.0 nm, while PVA/CMC/EM averaged 238.9 ± 18.0 nm; The average size of the fibers was determined in 193.5 ± 20.0 nm and 234.9 ± 28.0 nm for PVA/CMC/ZnO and EM-loaded PVA/CMC/ZnO nanocomposites, respectively; The PVA/CMC/EM nanofibrous mat showed a high initial burst release of EM (58%) Incorporation of 3% w/w ZnO NPs decreased the initial burst release of EM; EM-loaded PVA/CMC/ZnO nanocomposites were effective against *S. aureus* and *E. coli.*	[164]
**Cellulose Acetate**				
AgNPs	10% w/w CA in acetone/water at 4/1 v/v	Single nozzle electrospinning; AgNPs were added to CA solution at 0.0, 0.75 and 1.50% w/w; Fibers were produced using potential of 15 kV, distance of 10 cm and a feed rate of 3.0 mL/h;	Fiber diameters increased with increasing content of AgNPs, from ≈ 568 nm (pure CA) to ≈ 614 nm (1.50% w/w).	[167]
Titanium dioxide (TiO_2_)/AgNPs	17% w/v CA in DMF/acetone at 1/2 v/v	Single nozzle electrospinning; TiO_2_/AgNPs production: (1) 2/1% w/v DOPA in 1M Tris HCl buffer were used to coat TiO_2_ NPs; (2) DOPA-coated TiO_2_ were then added to 0.2 M AgNO_3_ and stirred for 18 h; (3) TiO_2_/AgNPs nanocomposite particles were centrifuged and dried at 60 °C for 12 h; 5% and 10% w/w TiO_2_/AgNPs were added to CA; Fibers were produced using potential of 15 kV and distance of 15 cm.	TiO_2_/AgNPs nanocomposite particles had spherical and rod-like shapes and sizes between 20 and 100 nm (average of ≈ 36.12 nm); As the NPs content increased so did the fibers diameters; Both studied NPs concentrations showed good antibacterial activities against *E. coli* and *S. aureus*.	[170]
ZnO/AgNPs	17% w/w CA in DMF/acetone at 1/2w/w	Single nozzle electrospinning; 5% and 10% w/w ZnO/AgNPs were mixed with CA; Fibers were produced using potential of 15 kV and distance of 15 cm.	CA, CA/ZnO and CA/ZnO/AgNP nanofibers were regular and bead free; Addition of AgNPs to CA/ZnO reduced the fibers diameters; CA/ZnO/AgNPs nanofibers were effective against *E. coli* and *S.* *aureus* bacteria; Nanocomposites containing 10% w/w ZnO/AgNPs yielded 0% viable bacteria cells in relative cell viability experiments.	[172]
Ag/Cupper (Cu) loaded onto sepiolite (SEP) and mesoporous silica	9% w/w CA in acetone/dH_2_O at 80/20 v/v	Single nozzle electrospinning; Two NPs were produced: NPs of silica SBA-15 contained 8.9% w/w Cu and 3.5% w/w Ag, and raw SEP NPs containing 24.4% w/w Ag and 18.5% w/w Cu; 5% w/w particles (in relation to the polymer and NPs mass) were added to CA; Fibers were produced using potential of 23 kV, distance of 15 cm and feed rate of 0.8 mL/h.	NPs became entrapped within the fibers during production; NPs were found well dispersed with occasional aggregates randomly distributed along the fibers; Diameters varied between 400 and 500 nm; All metal-loaded CA nanocomposites impaired significantly the growth of *Aspergillus niger;* The amount of metal NPs released daily by the nanocomposite represented ≈ 1% of the total amount of Ag or Cu.	[174]
Ag ions/AgNPs	10% w/w CA in acetone/water at 80/20 w/w	Single nozzle electrospinning; 0.0, 0.05, 0.30 and 0.50% w/w AgNO_3_ were added to CA; Fibers were produced using potential of 17 kV, distance of 10 cm and feed rate of 3 mL/h; Silver ions on the electrospun CA fibers were submitted to UV irradiation (photoreduction).	Fiber diameters decreased with AgNO_3_ increased content; Silver ions in ultrafine CA fibers were successfully photoreduced into AgNPs; The average diameters of the AgNPs were in the range of 3–16 nm; Both AgNO_3_ (non-reduced) and AgNPs (photoreduced) ultrafine CA fibers showed very strong antimicrobial activity.	[203]
**Cellulose Nanocrystalline**				
ZnO	10 % w/v PHBV in chloroform/DMF at 90/10 v/v	Single nozzle electrospinning; CNCs were prepared by acid hydrolysis in 9/1 v/v C_6_H_8_O_7_/ HCl at 80 °C for 6 h; Zn (NO_3_)_2_6H_2_O were added at ½ into CNCs; NaOH was added drop-wise to precipitate Zn^2+^; 0, 3, 5, 10 and 15 w/w% CNC/ZnO to PHBV and mixed for 24 h prior to spinning; Fibers were produced using potential of 18 kV, distance of 16 cm and feed rate of 1 mL/h.	Fiber diameters became narrower with higher loads of CNC/ZnO; The uniformity and porosity of the mats also increased with the higher incorporation of CNC/ZnO; The tensile strength and Young’s modulus were the most important with 5 w/w% CNC/ZnO; Mats with 5 w/w% CNC/ZnO had the highest water absorbency and exhibited the best antibacterial activity.	[186]
AgNPs	6% w/v PVA in dH_2_O	Single nozzle electrospinning; Synthesis of CNCs: (1) cellulose-rich cotton fibers were immersed in a NaOH solution (2% w/v) to remove impurities; (2) samples were hydrolyzed in HCl; CNCs were surface modified with succinic anhydride (SA) for 24 h; Modified CNCs (0.5 g) and AgNO_3_ at 0.05 M were mixed for 15 h, filtered and washed, and finally added to PVA; Fibers were produced using potential of 15 kV, distance of 15 cm and feed rate of 0.3 mL/h.	Films were smooth, highly flexible and displayed a highly homogeneous appearance; AgNPs coupled to the CNC were more effective against *P. aeruginosa.*	[204]
	16.6% w/w PVP in DMF	Single nozzle electrospinning; Synthesis of CNCs: CNCs were isolated from corn stalk using 60 w/w% sulfuric acid hydrolysis and mechanical treatments; AgNO_3_ and freeze-dried CNCs were dispersed in PVP at continuous stirring for 24 h at RT; Prepared samples: pure PVP, PVP/CNC-2%, PVP/CNC-4%, PVP/AgNO_3_-0.17%, PVP/AgNO_3_-0.34%, PVP/CNC-2%/AgNO_3_-0.17%, and PVP/CNC-2%/AgNO_3_-0.34% suspensions; Fibers were produced using potential of 18 kV, distance of 20 cm and feed rate of 1 mL/h.	Fiber diameters were the smallest for PVP/CNC-4%/AgNO_3_-0.34% (131 ± 46 nm); Upon addition of 4 w/w% CNCs, the ultimate tensile strength of pure PVP increased 0.8 MPa; PVP/CNC-4%/AgNO_3_-0.34% composites acted as excellent antimicrobial agents against both *E. coli* and *S. aureus.*	[205]
**Bacterial Cellulose (BC)**				
Soy protein NPs	5% w/v BC in TFA	Single nozzle electrospinning; Fibers were produced using potential of 30 kV, distance of 20 cm and feed rate of 0.2 mL/h; Surface functionalization: (1) 2.5% w/v of soy protein was dispersed in dH_2_O; (2) BC electrospun nanofiber scaffolds were immersed in soy protein solution and ultrasonicated for 1 h at 300 W for ultrasound-induced self-assembly process; (3) nanofibers were washed three times with ethanol/water mixture (70/30, v/v) to remove free soy protein molecules.	Nanofibers had a multi-size distribution with diameters ranging from 80 to 360 nm; After soy protein surface modification, nanofibers became more stretchable, increasing the elongation at break; Nanofibrous with soy protein NPs showed superior biocompatibility compared to pure BC electrospun nanofibers.	[190]
GO	3% w/v chitosan (CS) in acetic acid solution and 5% w/v BC prepared at 1/1, 4.5/1 and 8/1; 5% w/v PEO was added to the mixtures at different amounts	Single nozzle electrospinning; 0, 3, 6 and 10 v/v% PEO were added to CS/BC; PEO/CS/BC fibers were produced using potential of 20 kV, distance of 12 cm and feed rate of 0.3 mL/h; 0, 0.5, 1, 1.5 and 2 w/w% GO were added to CS/BC; GO/CS/BC fibers were produced using potential of 22 kV, distance of 10.	Mats with uniform morphologies were attained with 1.5% GO, however with 2% GO smaller diameters were generated; High amounts of GO increased the scaffold mechanical strength; A reduction in the hydrophilicity of the electrospun nanofibers and their water vapor permeability with the addition of GO was also reported.	[191]

Abbreviations - EM: erythromycin; AlCl_3_: aluminum chloride; DMF: dimethylformamide; AgNO_3_: silver nitrate; DOPA: dopamine hydrochloride; HCl: hydrochloric acid; PHBV: poly(3-hydroxybutyrate-co-3-hydroxy-valerate); C_6_H_8_O_7_: citric acid; *PVA:* poly(vinyl alcohol); EM: erythromycin; PNIPAAm: poly(N-isopropylacrylamide); PVP: poly(vinyl pyrrolidone); TFA: trifluoroacetic acid.

**Table 7:** Processing of cellulose-, CA-, and nanocellulose-containing electrospun mats incorporated with natural products.

Natural Extracts	Polymer Concentration/Ratio/Solvent	Incorporation of Agent and Production Conditions	Results	Ref.
**Cellulose**				
Bromelain	15% w/w CA in acetone/DMF at 85/15 w/w 15% w/w CTAc in acetone/DMF at 85/15 w/w 15% w/w 70%CA + 30%CTAB in acetone/DMF at 85/15 w/w	Single nozzle electrospinning; CTA was produced from CTAc and CTAB through traditional acetylation process with H2SO4 and C4H6O3; 0.0264 g of bromelain were added to 15% w/w 70%CA + 30%CTAB in acetone/DMF; Fibers were produced using potential of 25 kV, distance of 10 cm and feed rate of 4 mL/h; Bromelain was also immobilized via crosslinking on control fibers by immersion in 3-aminopropyl triethoxysilane and 1% v/v glutaraldehyde.	The acetyl content of CA was 41.9%, which corresponded to a D of 2.8; CTAC and CTAB solutions could not be electrospun because of their improper molar mass; CA fibers reached diameters of 470–755 nm and the CA+CTAB of 93–206 nm; Nanofibers immersed in a solution mimicking basic sweat had the lowest mass loss rate, not exceeding 9%, while in acid solutions they had the highest, ≈28%; *In vitro* controlled release tests were performed to semi-quantitatively evaluate the release profile of bromelain, which was completed in 3 days; Crosslinking was more effective than pos-electrospinning immobilization.	[165]
**Cellulose acetate**				
Cinnamon (CN); Lemongrass (LG); Peppermint (PM)	15% w/v CA in acetone	Single nozzle electrospinning; 5% v/v of selected EO in CA solution; Fibers were produced using potential of 15 kV, distance of 15 cm (maintained for all combinations) and feed rate of 5 mL/h for pristine CA, 25 kV and 3 mL/h for CA/CN, and 20 kV and 5 mL/h for both CA/LG and CA/PM.	The produced fibers were smooth, with diameters averaging ≈ 4.2 μm for CA, ≈ 0.9 μm for CA/CN, ≈ 2.8 μm for CA/LG and ≈ 2.3 μm for CA/PM; Fibers encapsulating 6.2 to 25.0% w/w of EOs were able to effectively stop proliferation of *E. coli*; EOs loaded mats were only effective against *C. albicans* with concentrations above 40% w/w; No cytotoxic effects were observed against fibroblasts and human keratinocyte cell lines.	[168]
Rosemary; Oregano	15% w/v of CA in acetone	Single nozzle electrospinning; 5% v/v of selected EO in CA solution; Fibers were produced using potential of −120 kV, distance of 15 cm and feed rate of 2 mL/h.	Fibers loaded with EOs revealed larger diameters because of the solution increased viscosity; Oregano oil was more effective than rosemary oil against bacteria; Rosemary oil was more efficient against the yeasts C. albicans than oregano oil.	[67]
Thymol (THY)	Porous mats: 5.75% w/v CA in acetone/DCM at 1/4 v/v; Nonporous mats: 15% w/w CA in acetone/DMAc at 3/2 v/v	Single nozzle electrospinning; Porous and nonporous mats: 0, 5, 10 and 15% w/w of THY (in relation to the polymer mass) mixed in the CA solution; Fibers were produced using potential of 18 kV, distance of 15 cm and feed rate of 2 mL/h.	Fibers from porous CA mats attained diameters of 2.95–4.66 μm; Fibers from nonporous CA mats exhibited smooth surface morphologies with diameters ranging 450–850 nm; Porous THY-loaded mats had a slower initial EO release, prolonging it over time, and reveling a superior antibacterial activity and cytocompatibility compared with the nonporous THY-loaded mats.	[65]
Zein; Streptomycin sulfate	15% w/w CA and 10% w/w polyurethane (PU), at 1/1, 2/1 and 3/1 v/v, in DMF/MEK at 50/50 w/w	Single nozzle electrospinning; 2% w/w of zein and 1% w/w of streptomycin sulfate were added to the CA/PU solutions; Fibers were produced using potential of 18 kV, distance of 15 cm and feed rate of 0.5 mL/h.	1/1 and 2/1 CA/Pu ratios registered bead formations on the surface; At 3/1 CA/PU fibers were more uniform exhibiting diameters of 400–700 nm; Loaded CA/PU accelerated blood clotting and enhanced fibroblasts growth, while displayed excellent bactericidal activity against *Bacillus subtilis* and *E. coli* bacteria.	[177]
Asiaticoside in the form of pure substance (PAC) and crude extract (CACE)	17% w/v CA in acetone/DMAc at 2/1 v/v; For comparison purposes, films were also produced by solvent-casting at 4% w/v CA in acetone/DMAc at 2/1 v/v	Single nozzle electrospinning; 40% w/w of PAC or CACE (in relation to the polymer mass) were added to the CA solutions, both for electrospinning or solvent-casting; Fibers were produced using potential of 17.5 kV, distance of 15 cm and feed rate of 1 mL/h.	Produced fibers were smooth even with the addition of the plant extracts; The average fiber diameter increased from 485 nm for PAC loaded to 545 nm for CACE loaded spun mats; Loaded electrospun mats showed higher capacity to retain water and resist weight loss than those films produced by solvent casting; All extract-loaded films were nontoxic to cells, the only exception being the highest concentration of CACE which was seen to lower cell viability.	[178]
Curc	17% w/v CA in acetone/DMAc at 2/1 v/v; For comparison purposes, films were also produced by solvent-casting at 4% w/v CA in acetone/DMAc at 2/1 v/v.	Single nozzle electrospinning; 5, 10, 15 and 20% w/w of Curc (in relation to the polymer mass) were added to the CA solutions, both for electrospinning and solvent-casting; Fibers were produced using potential of 17.5 kV, distance of 15 cm and feed rate of 1 mL/h.	Curc loading did not affect the electrospun mats morphology; The fiber diameter of Curc loaded CA fibers averaged 314–340 nm; The Curc loaded nanostructured mats antioxidant activity was superior to the casted films; Presence of Curc decreased cell viability but was not significant to pose any threats to the normal function of the human dermal fibroblast.	[179]
	10% w/w CA in acetone/water at 80/20 v/v; 10% w/w polyvinylpyrrolidone (PVP) in acetone/water at 50/50 v/v; 10% w/w CA/ PVP in acetone/water at 70/30 v/v.	One-pot electrospinning using the dual spinneret technique; 10% w/w of Curc (in relation to the polymer mass) were added to the CA, PVP or CA/PVP solutions; Fibers were produced using potential of 25 kV, distance of 15 cm and feed rate of 3 mL/h.	Diverse fiber diameters were obtained: ≈ 780 nm for neat CA, ≈ 495 for neat PVP, ≈ 1150 for Curc/CA, ≈ 570 for Curc/PVP, and ≈ 1560 for Curc/CA/PVP; Incorporation of PVP increased the fibers hydrophilicity and accelerated Curc release; Mats prepared by dual-spinneret electrospinning, namely Curc/CA+Curc/PVP, exhibited the highest antibacterial activity against *S. aureus*.	[26]
Asiaticoside in form of PAC and CACE; Curc	17% w/v CA in acetone/DMAc at 2/1 v/v.	Single nozzle electrospinning; 5, 10, 15 and 20% w/w of Curc (in relation to the polymer mass) were added to the CA solutions; 2, 40% w/w of PAC or CACE (in relation to the polymer mass) were added to the CA solutions; Fibers were produced using potential of 17.5 kV, distance of 15 cm and feed rate of 1 mL/h.	As-loaded herbal mats remain stable up to 4 months of storage, either at RT or 40 °C; Curc loaded mats showed superior antioxidant capacity compared to PAC or CACE containing mats; PAC and CACE loaded structures were more biocompatible the Curc loaded counterparts; 40% w/w PAC loaded surfaces supported the most attachment and proliferation of fibroblasts; Higher syntheses of collagen was observed for cells cultured on CA fibers that containing either 2% w/w CACE or 40% w/w PAC.	[210]
Gallic acid (GA)	17% w/v CA in acetone/DMAc at 2/1 v/v	Single nozzle electrospinning; 2.5–10% w/w of GA (in relation to the polymer mass) were added to the CA solutions; Fibers were produced using potential of 12 kV, distance of 12.5 cm and feed rate of 0.1 mL/h.	Fiber diameters increased linearly with the amount of GA; GA aggregation of GA was observed on surfaces loaded with 7.5–10% v/v GA; GA was successfully released from the electrospun mats.	[180]
Gingerol	12% w/v CA in acetone for 2 h at 25 °C; For comparison purposes, films were also produced by solvent-casting at 12% w/v CA in acetone	Single nozzle electrospinning; 6% w/w of gingerol were added to the CA solutions, both for electrospinning and solvent-casting; Fibers were produced using potential of 7.5 kV, distance of 10 cm and feed rate of 0.7 mL/h.	Fibers were smooth, varying from ≈ 475 nm (pristine) to 375 nm (loaded) in diameter, and with a very small number of beads being detected; ≈ 97% of the loaded gingerol could be released from the fibers at 37 °C; The release rate of gingerol increased drastically in the first 4 h (≈ 92%) and remained constant after that period; 2,2-diphenyl-1-picrylhydrazyl (DPPH) scavenging assays and in vitro cytotoxicity tests showed the antioxidant activity of the prepared fibers and a viability above 60% for L-929 mouse fibroblast-like cells.	[181]
Garlic extract	9.6% w/v CA and 9% w/v PVP in 98% acetic acid	Single nozzle electrospinning; Garlic extraction: (1) the garlic was crushed and macerated in ethanol at 1/1 w/w for two nights at 4 °C; CA solution was mixed with PVP at 8:5, which made the ratio of the dry weight of PVP to CA of 3/2; For every 13 g of PVP/CA 1 g of glycerine was added (PVP/CA/glycerine) or 1 g of garlic extract (PVP/CA/garlic); combinations of the two were also made; Fibers were produced using potential of 15 kV and distance of 12 cm.	The composite nanofibrous mats were uniform, bead-free with a size ranging from 350 nm to 900 nm; Release of garlic extract from PVP/CA/glycerine/garlic was the most important due to the large diameter of the fibers; The antibacterial activity of the PVP/CA/garlic nanofibrous mat was effective against both *S. aureus* and *P. aeruginosa*; PVP/CA/glycerine/garlic fibers were the most antimicrobial.	[182]
**Cellulose Nanocrystalline**				
Thymol	9% w/v PVA in dH2O	Single nozzle electrospinning; 30% w/w CNCs (in regard to PVA concentration) were prepared in dH2O/H2SO4 and added to PVA; Fibers were produced using potential of 10 kV, distance of 10 cm and feed rate of 0.25 mL/h. Electrospun PVA/CNCs was mixed with PLA in CHCl3 to obtain blends with a final concentration of 1 % w/w; nanocomposite films were impregnated with thymol dissolved in supercritical carbon dioxide (scCO2).	PVA/CNCs nanofibers impregnated with thymol registered a yield of 20%, while the PLA films obtained 24%; The release rate of thymol was significantly slower when PVA/CNCs were incorporated within a PLA matrix.	[187]
**Bacterial Cellulose**				
Tragacanth gum (TG)	7.7% w/w of keratin/PEO at 70/30 in dH2O;	0, 1, 3 and 5% w/w of BC were added to the keratin/PEO solution; Fibers were produced using potential of 22 kV, with distance of 10 cm and feeding rate of 0.1 mL/h; TG was incorporated by electrospraying as the nanofibers were being electrospun.	The mean fiber diameter of the mats composed by keratin/PEO was 243 ± 57 nm and reduced to 150 ± 43 nm with the addition of 1% or higher % of BC; BC (1%) significantly reduced the hydrophobicity of the mat; TG and BC modified mats promoted cell attachment and proliferation on the surface of the nanofibers.	[192]

Abbreviations - CTA: cellulose triacetate; CTAc: commercial cellulose; CTA_B_: sugarcane bagasse cellulose; H_2_SO_4_: sulfuric acid; C_4_H_6_O_3_: acetic anhydride; EO: essential oil; DCM: dichloromethane; DMAc: dimethylacetamide; DMF: N,N-dimethylformamide; MEK: methylethylketone/2-butanone; PVA: poly(vinyl alcohol); CHCl_3_: chloroform; TEC: triethyl citrate; PLGA: poly(lactic-co-glycolic) acid; HFIP: hexafluoroisopropanol; PEI: polyethyleneimine; CMCS: carboxymethyl chitosan; ANG: angiogenic factor.
